# The impact of COVID-19 on sexual risk behaviour for HIV acquisition in east Zimbabwe: An observational study

**DOI:** 10.1371/journal.pgph.0003194

**Published:** 2024-07-17

**Authors:** Rebekah Morris, Simon Gregson, Rufurwokuda Maswera, Louisa Moorhouse, Tawanda Dadirai, Phyllis Mandizvidza, Brian Moyo, Owen Mugurungi, Constance Nyamukapa

**Affiliations:** 1 Department of Infectious Disease Epidemiology and MRC Centre for Global Infectious Disease Analysis, School of Public Health, Imperial College London, London, United Kingdom; 2 Manicaland Centre for Public Health Research, Biomedical Research and Training Institute, Harare, Zimbabwe; 3 AIDS and TB programme, Zimbabwe Ministry of Health and Child Care, Harare, Zimbabwe; Qatar University College of Medicine, QATAR

## Abstract

The Covid-19 pandemic and associated restrictions have the potential to alter sexual risk behaviours for HIV acquisition with important implications for HIV prevention programmes in sub-Saharan Africa. To date, no large-scale data have been published to substantiate hypothesised changes in sexual risk behaviours. We used longitudinal survey data to assess the impact of Covid-19 on sexual risk behaviours in east Zimbabwe. Data on sexual behaviours in HIV-negative adults aged 15–54 years were collected in two rounds of a general population open-cohort survey conducted in Manicaland, Zimbabwe shortly before (July 2018 to December 2019; N = 7316) and several months into the Covid-19 epidemic (February to July 2021; N = 6356). Descriptive statistics and logistic regression models of serial cross-sectional and prospective cohort data were used to assess changes in sexual risk behaviours. The proportion of females aged 15–19 years reporting sexual debut declined from 29.7% before Covid-19 to 20.3% during Covid-19 (adjusted odds ratio (AOR) = 0.49, 95% confidence interval (95% CI), 0.38–0.63). Fewer sexually-active females reported multiple sexual partners during Covid-19 (3.35% *versus* 6.07%; AOR = 0.55, 95% CI, 0.43–0.72). No population-level changes in male behaviour between survey rounds were recorded but the cohort analysis revealed a complex pattern of behaviour change with HIV risk behaviours increasing for some individuals and decreasing for others. Overall HIV risk behaviours remained high in a sub-Saharan African population with a generalised HIV epidemic over a period of Covid-19 lockdowns when movements and social contacts were restricted.

## Introduction

The Covid-19 pandemic and associated government and community responses, through their socio-economic impacts and effects on national healthcare services, had the potential to have disastrous consequences in reversing previous gains in tackling the HIV epidemic [1]. In response to Covid-19, from early 2020, governments across the globe implemented regulations to restrict movement and social mixing in the hope of controlling the spread of SARS-CoV-2 infection. The restrictions aimed to prevent the spread of an airborne virus but could also have affected the spread of a sexually transmitted virus such as HIV. This is because they could have altered sexual behaviours which are key drivers of the spread of HIV infection within populations [2]. Contextual factors that contribute to Sexual Risk Behaviours (SRBs), including alcohol use and attendance at beer halls [3–5], are also likely to be impacted by mobility restrictions and a changing economic situation. Examples of SRBs for HIV acquisition include early age at first sex [6, 7], multiple serial or concurrent sexual partners [8, 9] and disassortative sexual mixing patterns [10, 11]. Changes in SRBs occurring within populations have altered the trajectory of HIV epidemics before. For example, declines in HIV infection rates in several countries in sub-Saharan Africa between 1990 and 2004 ‐ before Antiretroviral therapy (ART) was widely available ‐ were attributed in part to reductions in SRBs [12].

In sub-Saharan African countries with generalised HIV epidemics such as Zimbabwe, government lockdowns and other Covid-19 control restrictions have often been tight and the socio-economic impact of the Covid-19 pandemic has been far-reaching [13, 14]. However, no previous studies have measured the effects of the pandemic and associated responses on levels of SRB for HIV infection in the general population in these settings.

Much of the existing evidence is focused on men (cis and trans) and other gender-diverse people who have sex with men (MGDSM) populations in high-income countries with the general finding that risk behaviours, or sexual activity more broadly, declined dramatically early on in the Covid-19 pandemic in 2020 [15–17]. One study examined changes to sexual risk behaviour amongst the general population in Panama, with 28.8% of participants reporting a decrease in casual sex and 68.6% reporting no change [18].

To date, data from sub-Saharan African settings are limited to studies focusing on specific sub-populations. Davey and colleagues reported similar levels of sexual activity before and during lockdown amongst HIV-uninfected women in South Africa who completed surveys at their first antenatal visit [19]. Kavanagh and colleagues describe a reduction in sexual risk behaviours associated with Covid-19 in a cohort of women at high risk of HIV infection in rural Kenya [20]. Reported numbers of sexual partners, including transactional sex partners, declined and Covid-19 was associated with reductions in economic security. In contrast Sun and colleagues found a significant increase in transactional sex amongst adolescents in Botswana [21].

In the current article, we address this knowledge gap on the question of how Covid-19 affected SRB for HIV infection within the general population in sub-Saharan African countries during the first year of the pandemic through a case study of Manicaland, east Zimbabwe. Primary objectives of the study were: 1) to compare levels of SRB in representative cross-sectional samples of adults in the general population during and prior to Covid-19; 2) to describe changes in SRB in a cohort of adults followed from shortly before the outbreak of Covid-19 in Zimbabwe in March 2020 to the mid-point of the third (Delta) wave of the national epidemic in July 2021; and 3) to document the social impacts and responses to Covid-19 that could underpin changes in SRB. Sub-analyses were carried out to examine differences in reported social impacts of Covid-19 and SRBs at different time points in the pandemic. By limiting our analysis to HIV-negative individuals living in a population with high HIV prevalence we are able to focus on the population at risk of HIV acquisition.

## Methods

### Study setting

**Fig 1:**
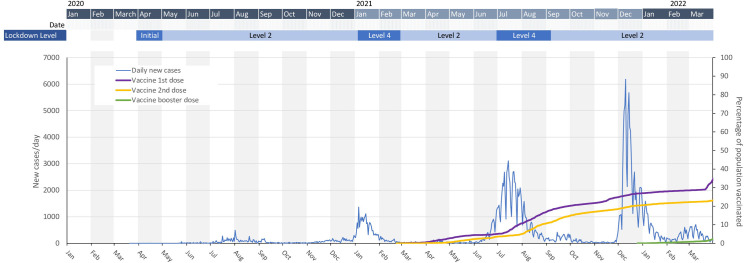
Trends in reported national COVID-19 infections and deaths, 2020 to 2022. January 2021 –Level 4 lockdown: Curfew 18:30–06:00, Only essential shops open, Intercity movement prohibited, Beer halls and Night clubs closed, bottle stores closed, public gatherings banned except funerals limited to 30 people. March 2021 –Level 2 lockdown: Curfew 22:00–05:30, All businesses to open with normal business hours, no restrictions on intercity travel, Beer halls and nigh clubs closed, Bottle stores takeaway only, Funeral gatherings to remain at 30 people other social gatherings limited to 50 people. June 2021 –Level 4 lockdown, curfew 18:30–06:00, commerce and industry open 08:00–15:30, intercity movement prohibited, Beer halls and night clubs closed, Bottle stores takeaway only, Public gatherings banned except funerals limited to 30 people.

In Zimbabwe, the government declared Covid-19 a national disaster on March 17, 2020 with public events and gatherings cancelled or postponed. The first case was identified on March 21, 2020 and multiple measures were quickly put in place to contain the spread of the SARS-CoV-2 virus (Fig 1). These measures included bans on public gatherings such as international sporting fixtures, church services and weddings and closures of schools [22]. On March 30, 2020, Zimbabwe went into national lockdown. Subsequently, with the emergence of progressively more virulent variants [23], the country experienced four major waves of infections with cases peaking in August 2020, January 2021, July 2021 and December 2021. The national vaccination programme began in February 2021 [24].

The study was conducted in eight sites in Manicaland, east Zimbabwe, representing urban, peri-urban, agricultural estate and subsistence farming areas in the province. These included six of the twelve sites in which reductions in HIV infection rates and associated SRBs were recorded in earlier rounds of a general population open-cohort survey [25]. The demographics of the study population have been well documented in a number of previous publications [25, 26]. HIV prevalence was 11.3% and ART coverage was 65.0% in the study population (ages 15 years and above) shortly before the Covid-19 pandemic [26].

### Data source

Data for the study were taken from two rounds of a general population open-cohort survey in Manicaland conducted shortly prior to (July 2018 to December 2019) and several months following the outbreak of the Covid-19 epidemic (February to July 2021) in Zimbabwe. As new, as well as pre-existing, participants were recruited based on an updated population census at the start of each round, it can be described as an open-cohort survey. The second round started just after the President announced an easing of a strict ‘level 4’ lockdown (February 15, 2021) and schools closures (March 21, 2021) introduced to control the second (Beta variant, B.1.351) major wave in the national epidemic (Fig 1). By June 2021 –just over half-way through the survey period–the third wave of SARS-CoV-2 infections had hit, driven by the more transmissible Delta variant (B.1.617), with case numbers rising drastically. The government responded again by tightening restrictions, with local lockdowns introduced in May 2021 followed by a national ‘level 4’ lockdown from June 14, 2021.

In each round of the survey, an initial household census was carried out in each study site and individuals aged ≥15 years resident in the enumerated households were eligible and invited to participate in individual interviews. Participants for individual interviews were recruited between 6^th^ July 2018 and 19^th^ October 2019 for the first round used in this analysis and between 24^th^ February 2021 and 27^th^ July 2021 for the second. For the pre-Covid-19 survey, all younger people were eligible to participate but older people were eligible only if they were resident in a random sample of two-thirds of households.^26^ This random sampling allows for serial cross-sectional analysis of population-level changes occurring between the two survey rounds. Interviews were done face-to-face at participants’ households and provider-initiated HIV testing and counselling (PITC) was conducted. Participants who opted out of PITC were requested to provide dried blood spot (DBS) samples for laboratory testing.^26^ In the during-Covid survey, eligibility for the individual interview was restricted to those resident in the random sample of two-thirds of households for participants of all ages. This survey was limited by Covid-19 safety measures with interview procedures adapted to take place over the telephone and self-reported data collected on HIV testing and infection status.

### Ethics statement

Ethical approval for the study was obtained from the Medical Research Council of Zimbabwe (MRCZ/A/2703) and the Imperial College Research Ethics Committee (20IC6436). All participants provided informed consent prior to participation in the study. For participants under the age of 18 informed consent was obtained from their parent/guardian in addition to participant assent. Consent was obtained verbally, recorded, with audio files stored securely and separate from the main study dataset.

### Inclusivity in global research

Additional information regarding the ethical, cultural, and scientific considerations specific to inclusivity in global research is included in S1 Checklist.

### Study measures

In the pre-Covid-19 survey round, HIV infection status was established using results from PITC and, where PITC results were not available and informed consent was given, from laboratory testing [26]. In the during-Covid-19 round, HIV status infection was determined using test results from the pre-Covid-19 survey round updated with self-reports: 1) for individuals who participated and tested HIV-negative in the pre-Covid-19 round, of results from any HIV testing received between rounds; or 2) for individuals newly enrolled in the survey, of results of their most recent HIV test. Participants in the during-Covid-19 survey round who had never had an HIV test or who were HIV-negative in the pre-Covid-19 round and had not had a test since then were assumed to be uninfected.

Data on participants’ demographic characteristics (age-group, marital status, education–including school enrolment and attendance, employment and household wealth) and use of alcohol and recreational drugs were collected in the survey questionnaires (S2 Text and S1 Table). In the during-Covid-19 survey round, data were collected on awareness of Covid-19 and its symptoms (shortness of breath, sore throat, loss of sense of smell and taste), prevention behaviours (isolating, missing school and getting vaccinated), and perceived risk of SARS-CoV-2 infection (S2 Table).

In each round, data were collected on SRB variables including sexual debut for participants aged 15–19 years, and for those aged 15–54 years who had started sex: multiple sexual partners; concurrent partnerships; non-regular partners; transactional sex; and, for women only, having had an age-disparate sexual relationship. Data were also collected on symptoms of other sexually transmitted infections (S3 Table). Risk categories for HIV acquisition were created to assess overall changes in SRB. High, medium, and low risk categories were defined as having ≥1 non-regular partners in the past year; not high risk but having multiple partners in the past year or transactional sex in the past month with any of the 3 most recent partners; and not high or medium risk but having started sex, respectively (S2 Text).

### Data analysis

A serial cross-sectional period analysis, disaggregated by sex, was carried out to assess for changes in the socio-demographic profile of the HIV-negative study population between the two survey rounds due to Covid-19 or the changes in survey methods. For this, descriptive statistics (proportions and 95% confidence intervals) were calculated with proportions weighted to account for under-sampling of older age-groups in the pre-Covid-19 round. Logistic regression models were used to estimate adjusted odds ratios for each demographic characteristic. This analysis was repeated for the cohort of HIV-negative individuals who participated in both survey rounds.

Measurements of attitudes and experiences of Covid-19 amongst the HIV-negative population in the during-Covid-19 survey round were made using descriptive statistics. These included missing school due to Covid-19 (amongst 15–19 year-olds enrolled in school), awareness of Covid-19 symptoms, awareness of modes of transmission, and having relocated due to Covid-19, isolated due to Covid-19, worn a facemask outside, tested for Covid-19, been vaccinated against Covid-19, and perceiving risk of getting Covid-19.

Descriptive statistics and logistic regression analyses were carried out for changes in SRB over time (serial cross-sectional period analysis) and over time with increasing age (cohort analysis). These analyses were carried out for individual risk behaviours and for overall risk categories. Sankey diagrams were created using the cohort analysis to elucidate how participants’ overall risk categories changed between pre-Covid-19 and during-Covid-19 periods.

To explore the possible effects of changes in Covid-19 cases and control measures occurring within or accumulating over the period of data collection in the during-Covid-19 survey round, the data on attitudes and experiences of Covid-19 and SRB categories were divided into three time periods of data collection–Period 1: February and March 2021 (the tail end of the second wave of Covid-19 infections in Zimbabwe); Period 2: April and May 2021 (period of relatively low infection rates in Zimbabwe and easing of restrictions); and Period 3: June and July 2021 (when the Delta variant driven third-wave took hold and stricter restrictions were reinstated).

For the cross-sectional analyses, study participants were limited to those who were HIV-negative and aged 15–54 years at the time of each survey round. For the cohort analysis, participants were included if they were aged 15–54 years and HIV-negative at the time of the pre-Covid-19 round. Participants were excluded if they reported becoming infected with HIV between the survey rounds.

## Results

### Participation rates and participant characteristics

In the pre-Covid-19 survey round, 7609 households were identified and 7571 (99.5%) consented to the household interview (S1 Fig). From these households, 12692 individuals were eligible for the survey and 9803 (77.2%) participated. Of these individuals, 7316 met the inclusion criteria for this analysis. In the during-Covid-19 round, 7276 eligible households were identified and 6107 (83.9%) consented to participate (S2 Fig). 9383 household members were eligible for interview and 8497 (90.6%) participated. 6314 met the inclusion criteria for the analysis. For the cohort analysis, 4742 individuals participated in both survey rounds (follow-up rate, 75.2%; S3 Fig); 3168 of these were eligible for the cohort analysis.

After weighting to account for the changes in sampling between survey rounds, the mean ages of eligible participants in the pre-Covid-19 and during Covid-19 rounds were 29.4 years and 30.1 years, respectively. The proportions of males in each age-group in the two rounds were similar (Table 1). For females, there were fewer participants aged 20–24 years in the during-Covid-19 round (15.9%; 95% CI 14.7–17.1) compared to the pre-Covid-19 round (18.8%; 95% CI 17.7–19.9).

**Table 1:** Demographic characteristics of HIV negative adults aged 15–54 in Manicaland, prior to and during the Covid-19 pandemic.

	A) Males								B) Females							
	Cross-sectional analysis				Cohort analysis				Cross-sectional analysis				Cohort analysis			
	Pre-Covid	During Covid			Pre-Covid	During Covid			Pre-Covid	During Covid			Pre-Covid	During Covid		
	% (95%CI)*	% (95%CI)	AOR^†^ (95%CI)	p	% (95%CI)	% (95%CI)	AOR^†^ (95%CI)	p	% (95%CI)*	% (95%CI)	AOR^†^ (95%CI)	p	% (95%CI)	% (95%CI)	AOR^†^ (95%CI)	p
**15-54yrs**	**N = 3181**	**N = 2773**			**N = 1171**	**N = 1171**			**N = 4135**	**N = 3541**			**N = 1997**	**N = 1997**		
**Age Group**																
15–19	26.4 (25.0–27.9)	25.8 (24.2–27.5)			25.8 (23.4–28.4)	17.8(15.7–20.1)			21.5(20.4–22.7)	20.7 (19.4–22.1)			15.7 (14.2–17.4)	10.6 (9.34–12.0)		
20–24	17.6 (16.4–18.9)	16.1 (14.8–17.5)			12.7 (10.9–14.8)	15.5(13.6–17.7)			18.8(17.7–19.9)	15.9 (14.7–17.1)			14.6 (13.1–16.2)	12.8 (11.4–14.3)		
25–29	11.7 (10.7–12.8)	12.3 (11.1–13.5)			9.74 (8.16–11.6)	10.8 (9.19–12.8)			13.8(12.7–15.0)	14.5(13.4–15.7)			13.8(12.3–15.4)	15.2(13.7–16.8)		
30–34	12.3 (11.1–13.7)	10.4 (9.34–11.6)			11.5 (9.82–13.5)	10.6 (8.95–12.5)			13.7(12.6–14.9)	12.5(11.4–13.6)			15.7(14.1–17.3)	13.9 (12.5–15.5)		
35–44	20.2 (18.7–21.8)	21.8 (20.3–23.4)			24.4 (22.0–27.0)	25.7(23.3–28.3)			20.2(18.9–21.5)	22.7 (21.3–24.1)			25.2 (23.3–27.1)	27.6 (25.7–29.6)		
45–54	11.8 (10.6–13.1)	13.6 (12.3–14.9)			15.8 (13.8–18.0)	16.5(14.5–18.7)			12.0(10.9–13.1)	13.7 (12.6–14.9)			15.0 (13.5–16.7)	16.4 (14.9–18.1)		
**55+**						3.07(2.22–4.23)								3.51 (2.78–4.41)		
**Site Type** ^**‡**^																
Urban	14.5 (13.3–15.7)	20.6 (19.1–22.1)	1.53 (1.33–1.75)	<0.001	14.8 (12.9–16.9)	14.8(12.9–16.9)	0.99(0.78–1.25)	0.93	18.7(17.6–20.0)	19.5 (18.2–20.8)	1.06 (0.95–1.19)	0.32	15.6 (14.0–17.2)	15.6 (14.0–17.2)	1.04 (0.87–1.23)	0.69
Periurban	23.1 (21.6–24.6)	27.8 (26.1–29.5)	1.28 (1.14–1.44)	<0.001	25.7 (23.3–28.3)	25.7(23.3–28.3)	1.03(0.85–1.24)	0.75	28.1(26.7–29.5)	30.3 (28.8–31.9)	1.12 (1.01–1.24)	0.03	30.2 (28.3–32.3)	30.2 (28.3–32.3)	1.01 (0.88–1.16)	0.84
Estates	32.6 (30.9–34.3)	24.8 (23.3–26.5)	0.68 (0.61–0.77)	<0.001	30.3 (27.7–33.0)	30.3(27.7–33.0)	1.00(0.83–1.20)	0.99	21.9(20.7–23.3)	21.2 (19.9–22.6)	0.95 (0.85–1.06)	0.34	21.1 (19.4–23.0)	21.1 (19.4–23.0)	0.98 (0.84–1.14)	0.78
Rural	29.9(28.3–31.5)	26.8 (25.2–28.5)	0.86 (0.76–0.96)	0.01	29.2 (26.7–31.9)	29.2(26.7–31.9)	0.98(0.82–1.17)	0.81	31.2(29.8–32.7)	29.0 (27.5–30.5)	0.89 (0.81–0.99)	0.03	33.0 (31.0–35.1)	33.0 (31.0–35.1)	0.98 (0.86–1.12)	0.79
**Marital Status**																
Never married	43.7 (42.0–45.5)	43.3 (41.5–45.2)	1.08 (0.91–1.29)	0.36	38.4 (35.7–41.3)	33.7(31.1–36.5)	1.13(0.83–1.55)	0.44	23.8 (22.6–25.1)	23.9 (22.5–25.3)	1.17 (1.02–1.35)	0.03	17.7 (16.1–19.4)	15.2 (13.7–16.9)	1.34 (1.05–1.71)	0.02
Married/cohabiting	52.5 (50.8–54.3)	53.4 (51.5–55.3)	0.97 (0.83–1.14)	0.69	59.1 (56.2–61.9)	62.9(60.0–65.6)	0.83(0.63–1.10)	0.20	64.8 (63.3–66.2)	63.0 (61.4–64.6)	0.85 (0.76–0.95)	0.003	71.6 (69.6–73.5)	73.1 (71.1–75.0)	0.92 (0.78–1.08)	0.30
Divorced/separated	3.51 (2.88–4.28)	2.92 (2.36–3.62)	0.86 (0.63–1.17)	0.34	2.22 (1.52–3.24)	3.07(2.22–4.23)	1.29(0.77–2.17)	0.33	8.58 (7.73–9.52)	9.86 (8.92–10.9)	1.14 (0.97–1.33)	0.12	7.66 (6.57–8.91)	7.41 (6.34–8.65)	0.88 (0.69–1.13)	0.32
Widowed	0.23 (0.10–0.51)	0.36 (0.19–0.67)	1.35 (0.48–3.78)	0.57	0.26 (0.08–0.79)	0.34(0.13–0.91)	1.30(0.29–5.85)	0.73	2.80 (2.30–3.40)	3.28 (2.74–3.92)	1.10 (0.82–1.46)	0.53	3.05 (2.38–3.91)	4.26 (3.45–5.24)	1.04 (0.72–1.51)	0.83
**Household Wealth Index** ^**§**^																
Poorest	9.81 (8.79–10.9)	5.59 (4.80–6.51)	0.55 (0.45–0.68)	<0.001	9.65 (8.08–11.5)	7.34 (5.98–8.99)	0.72 (0.53–0.99)	0.04	8.97 (8.12–9.90)	6.70 (5.92–7.57)	0.74 (0.62–0.88)	0.001	9.51 (8.30–10.9)	8.17 (7.04–9.45)	0.84 (0.67–1.06)	0.15
2nd poorest	46.5 (44.8–48.3)	42.1 (40.3–44.0)	0.98 (0.88–1.10)	0.77	42.7 (39.9–45.6)	42.5 (39.7–45.4)	1.00 (0.84–1.19)	0.99	40.3 (38.8–41.9)	41.4 (39.8–43.1)	1.11 (1.00–1.22)	0.04	41.5 (39.4–43.7)	42.9 (40.7–45.1)	1.06 (0.93–1.22)	0.38
3rd poorest	22.3 (20.8–23.8)	29.3 (27.7–31.1)	1.41 (1.25–1.59)	<0.001	24.0 (21.6–26.5)	28.4 (25.9–31.1)	1.26 (1.04–1.52)	0.02	24.0 (22.7–25.4)	28.1 (26.7–29.6)	1.22 (1.10–1.36)	<0.001	24.3 (22.5–26.2)	26.2 (24.3–28.1)	1.11 (0.96–1.28)	0.17
4th poorest	20.1 (18.8–21.6)	22.2 (20.7–23.8)	0.90 (0.78–1.03)	0.12	22.1 (19.8–24.6)	20.9 (18.7–23.3)	0.91 (0.73–1.13)	0.40	25.0 (23.7–26.3)	22.7 (21.4–24.1)	0.81 (0.72–0.91)	<0.001	23.3 (21.5–25.2)	21.8 (20.0–23.7)	0.89 (0.76–1.05)	0.18
Least poor	1.25 (0.91–1.70)	0.76 (0.49–1.16)	0.52 (0.30–0.89)	0.02	1.54 (0.97–2.43)	0.77 (0.40–1.47)	0.91 (0.73–1.13)	0.40	1.70 (1.34–2.15)	1.02 (0.73–1.41)	0.58 (0.39–0.88)	0.01	1.40 (0.97–2.02)	1.00 (0.65–1.55)	0.74 (0.41–1.32)	0.31
**Employment**																
Professional or managerial	4.03 (3.35–4.85)	6.31 (5.46–7.28)	1.48 (1.15–1.91)	0.00	4.95 (3.85–6.36)	6.40 (5.14–7.96)	1.20 (0.83–1.73)	0.33	3.63 (3.06–4.30)	4.69 (4.04–5.44)	1.21 (0.95–1.54)	0.12	3.61 (2.87–4.52)	4.61 (3.77–5.62)	1.20 (0.87–1.66)	0.27
Self-employed: small business	1.17 (0.83–1.63)	1.33 (0.97–1.84)	0.96 (0.60–1.55)	0.87	1.20 (0.71–2.01)	1.28 (0.77–2.11)	1.03 (0.49–2.15)	0.95	0.46 (0.29–0.76)	1.47 (1.12–1.92)	3.36 (1.90–5.93)	<0.001	0.60 (0.34–1.06)	1.60 (1.14–2.26)	2.48 (1.26–4.88)	0.01
Skilled labour	7.40 (6.48–8.43)	14.3 (13.1–15.7)	2.42 (2.01–2.92)	<0.001	8.28 (6.83–10.0)	15.8 (13.8–18.0)	1.96 (1.49–2.58)	<0.001	2.34 (1.90–2.89)	4.52 (3.88–5.25)	1.97 (1.50–2.58)	<0.001	2.40 (1.82–3.18)	3.76 (3.00–4.69)	1.47 (1.01–2.15)	0.04
Manual/unskilled labour	18.8 (17.5–20.3)	15.5 (14.2–16.9)	0.99 (0.84–1.16)	0.86	13.8 (12.0–15.9)	17.6 (15.5–19.9)	1.32 (1.03–1.69)	0.03	4.42 (3.80–5.13)	7.79 (6.96–8.72)	1.90 (1.55–2.34)	<0.001	4.36 (3.54–5.35)	8.21 (7.09–9.50)	2.00 (1.51–2.65)	<0.001
Informal employment	15.0 (13.7–16.3)	14.1 (12.8–15.4)	0.81 (0.69–0.94)	0.01	17.0 (14.9–19.3)	16.0 (14.0–18.2)	0.84 (0.67–1.06)	0.14	13.7 (12.6–14.8)	18.8 (17.6–20.1)	1.41 (1.24–1.60)	<0.001	15.9 (14.4–17.6)	23.6 (21.8–25.6)	1.56 (1.33–1.84)	<0.001
Student	22.2 (20.8–23.6)	19.8 (18.4–21.4)	0.77 (0.64–0.92)	0.004	24.2 (21.8–26.7)	11.7 (10.0–13.7)	0.32 (0.23–0.45)	<0.001	15.2 (14.3–16.3)	15.3 (14.2–16.6)	1.13 (0.96–1.32)	0.15	12.7 (11.3–14.2)	8.06 (6.95–9.34)	0.78 (0.58–1.04)	0.09
Unemployed	24.8 (23.3–26.4)	28.6 (27.0–30.3)	1.20 (1.06–1.35)	0.004	23.7(21.3–26.2)	31.3(28.7–34.0)	1.44 (1.18–1.74)	<0.001	52.5 (51.0–54.1)	47.4 (45.7–49.0)	0.82 (0.75–0.90)	<0.001	52.8 (50.6–55.0)	50.1 (47.9–52.3)	0.84 (0.73–0.95)	0.01
Other: unspecified^‖^	6.61 (5.76–7.57)				6.92 (5.60–8.52)				7.64 (6.84–8.53)				7.61 (6.53–8.86)			
**Alcohol/Drugs**																
Drank alcohol in the past year	26.9 (25.3–28.5)	21.7 (20.2–23.2)	0.73 (0.64–0.84)	<0.001	27.3 (24.8–30.0)	26.5 (24.0–29.1)	0.86 (0.71–1.05)	0.13	2.01 (1.62–2.50)	2.40 (1.94–2.96)	1.19 (0.87–1.62)	0.27	2.10 (1.56–2.83)	1.85 (1.35–2.55)	0.84 (0.53–1.33)	0.46
Visited bar, beerhall or shebeen in past month	31.0 (29.3–32.7)	16.9 (15.6–18.4)	0.44 (0.39–0.51)	<0.001	31.4 (28.8–34.1)	21.4 (19.1–23.9)	0.52 (0.43–0.63)	<0.001	0.62 (0.42–0.91)	0.62 (0.41–0.94)	0.97 (0.55–1.71)	0.91	0.40 (0.20–0.80)	0.60 (0.34–1.06)	1.42 (0.57–3.55)	0.45
Using drugs for pleasure	4.78 (4.08–5.61)	2.92 (2.36–3.62)	0.60 (0.45–0.79)	<0.001	3.76 (2.81–5.01)	3.67 (2.73–4.92)	0.90 (0.58–1.39)	0.63	0.19 (0.10–0.39)	0.34 (0.19–0.60)	1.81 (0.73–4.48)	0.20	0.05 (0.01–0.35)	0.30 (0.13–0.67)	6.50 (0.78–54.27)	0.08
**15-19yrs**	**N = 986**	**N = 716**			**N = 302**	**N = 302**			**N = 1112**	**N = 734**			**N = 314**	**N = 314**		
**Currently Enrolled in Education**	73.2 (70.4–75.9)	64.0 (60.4–67.4)	0.64 (0.50–0.82)	<0.001	84.4 (79.9–88.1)	37.7 (32.4–43.4)	0.33 (0.21–0.52)	<0.001	57.7 (54.8–60.6)	57.1 (53.5–60.6)	1.06 (0.84–1.33)	0.64	69.4 (64.1–74.3)	36.0 (30.8–41.5)	0.90 (0.59–1.37)	0.62

* In the cross-sectional analysis of the pre-covid survey, proportions are weighted to account for age bias in selection

† Odds ratios are adjusted for 5 year age group and site type. For variables limited to 15–19 year olds odds ratios are adjusted for site type and age in years.

‡ Note that no specific procedures were followed to identify individuals moving to different study site between the survey rounds

§Household wealth was estimated for each individual by assessing asset ownership within their household and attributing a score as described by Schur et al.19 Each asset variable was transformed into scores between 0 and 1. The values of each asset variable were then summed and divided by the total number of assets giving an overall score between 0 and 1. Equally spaced cut offs (0,0.2, 0.4, 0.6, 0.8) were used to categorise the overall wealth of a household into five groups.

‖ These individuals answered ’other’ for their category of employment but then did not specify in the follow up question so they could not be recoded to any of the other categories.

In the pre-Covid-19 round, the highest proportions of males were from the estates and the rural sites. These proportions were reduced in the during-Covid-19 rounds whilst the proportions living in urban and peri-urban sites both increased (Table 1). Similar trends were seen for females, but the percentage changes were smaller. In 15–19 year-old females, the proportion reporting never being married increased from 74.5% pre-Covid-19 to 82.4% during-Covid-19 (AOR 1.57, 95% CI 1.24–1.99). In the during-Covid-19 round, higher proportions of both male and female participants were from the middle wealth household group with fewer falling within the least poor and poorest categories.

### Social impacts and responses to Covid-19

**Fig 2:**
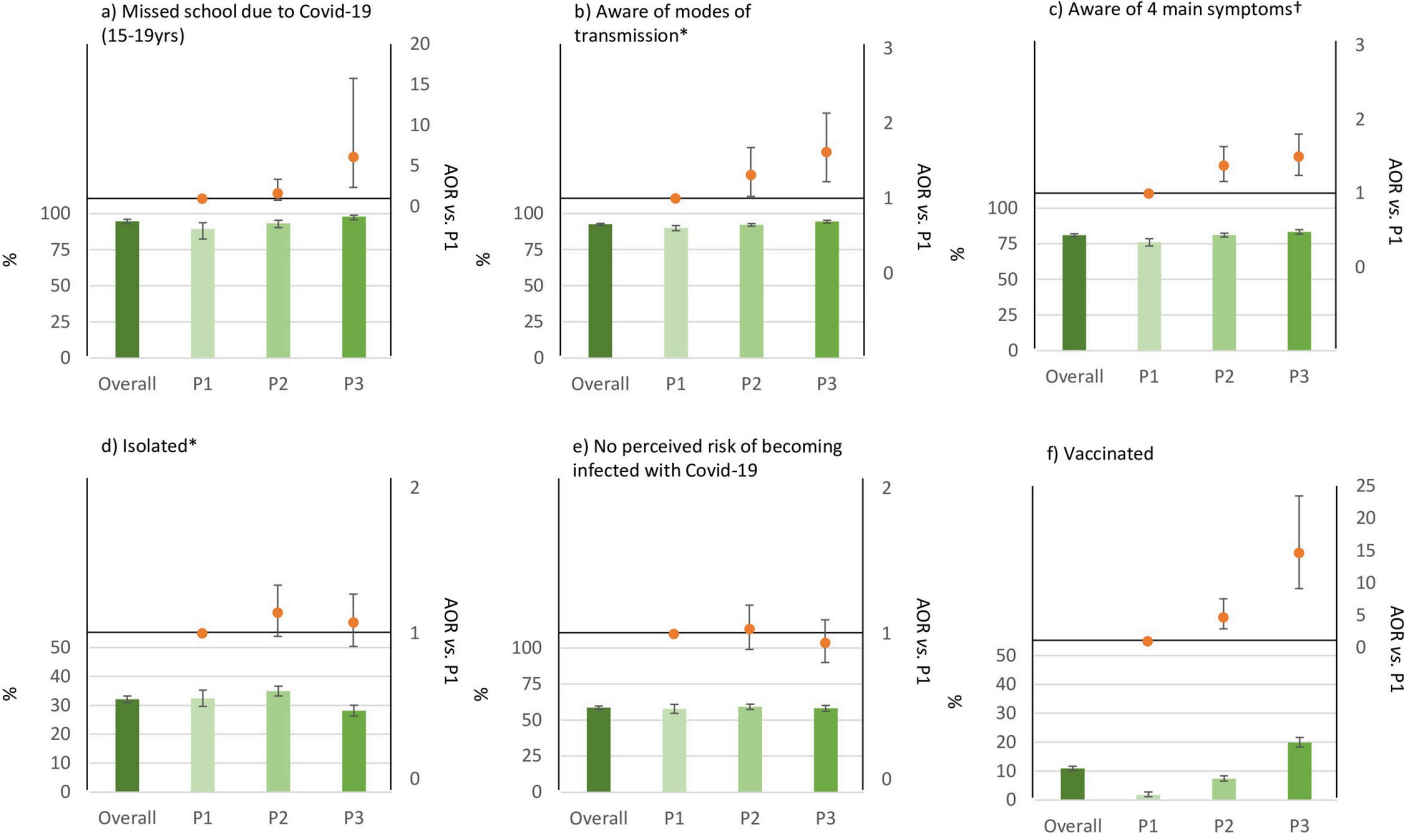
Population effects and responses to the Covid-19 pandemic amongst HIV negative adults aged 15–54 in Manicaland during three periods of the Covid-19 pandemic from February to July 2020 (P1: February-March 2020, P2: April-May 2020, P3: June-July 2020).

The proportion of study participants aged 15–19 years currently enrolled in school dropped substantially for males with no significant changes for females (Table 1). For 15–19 year-olds still enrolled in school, 94.3% and 95.2% of males and females, respectively, reported having missed school due to Covid-19; with the proportions particularly high in the third period of data collection in the during-Covid-19 round (Fig 2). Fewer males reported drinking alcohol at least once per month in the past year during Covid-19 than before Covid-19 (21.7% vs. 26.9%; AOR 0.73, 95% CI 0.64–0.84) but no change was found for females (Table 1).

Knowledge of the four main symptoms of Covid-19 was greater in females than in males (females: 85.3%; 95% CI 84.1–86.4; males: 75.8%; 95% CI 74.2–77.4) and fewer females than males perceived no risk of becoming infected with Covid-19 (females: 56.8%; 95% CI 55.1–58.4; males: 60.9%; 95% CI 59.0–62.8). Females were more likely to report no perceived risk of getting Covid-19 during period 2 of data collection compared to period 1 (AOR 1.22; 95% CI 1.01–1.48), corresponding with lower rates of infection and reduced government mandated restrictions.

Vaccination rates increased for both sexes over the data collection period; for females, the AOR of being vaccinated was 17.9 (95% CI 9.7–33.3) in period 3 compared with period 1 (S6 Table).

### Sexual risk behaviours

For males, in the serial cross-sectional analysis (Table 2), there was no evidence for changes in sexual debut. However, amongst 15–54 year-olds who had started sex, there was a trend towards fewer reporting multiple sexual partners in the past month during Covid-19 compared to before Covid-19 (4.74% vs. 5.93%; AOR = 0.77, 95% CI 0.58–1.02) which was statistically significant for males interviewed in the first period of the during-Covid-19 survey round (3.19% vs. 5.93%; AOR = 0.49, 95% CI 0.25–0.94; S7 Table). There was also a reduction in the proportion reporting STI symptoms in the past 12 months (1.42% vs. 3.12%; AOR 0.41, 95% CI 0.26–0.65) but no evidence for changes in any of the other HIV risk behaviours. In the cohort analysis, the proportion of males aged 15–19 years at the time of the pre-Covid-19 round who had started sex increased from 8.61% pre-Covid-19 to 32.5% during-Covid-19. For males aged 15–54 years who had started sex before Covid-19, after adjusting for differences in age and site type, there was a reduction in reports of recent STI symptoms but no other changes in SRBs (Table 2).

**Table 2:** Sexual risk behaviours of HIV negative adults aged 15–54 in Manicaland, prior to and during the Covid-19 pandemic.

	Cross-sectional analysis				Cohort analysis			
	Pre-Covid	During Covid			Pre-Covid	During Covid		
	% (95%CI)*	% (95%CI)	AOR^†^ (95%CI)	p	% (95%CI)	% (95%CI)	AOR^†^ (95%CI)	p
A) Males	**N = 3181**	**N = 2773**			**N = 1171**	**N = 1171**		
15-19yrs	**N = 986**	**N = 716**			**N = 302**	**N = 302**		
Had sexual debut (15-19yrs)†	10.8 (9.0–12.8)	13.0 (10.7–15.7)	1.25 (0.93–1.68)	0.38	8.61 (5.92–12.36)	32.5 (27.4–38.0)	5.15 (3.22–8.24)	<0.001
15-54yrs started sex	**N = 2050**	**N = 1963**			**N = 864**	**N = 864**		
Multiple partners in past 1 month	5.93 (4.97–7.06)	4.74 (3.88–5.77)	0.77 (0.58–1.02)	0.07	5.79 (4.41–7.56)	5.21 (3.91–6.91)	0.85 (0.56–1.30)	0.46
Multiple partners in past 12 months	17.8 (16.2–19.5)	17.9 (16.2–19.6)	1.01 (0.86–1.19)	0.91	15.7 (13.5–18.3)	14.6 (12.4–17.1)	0.99 (0.76–1.30)	0.95
Concurrent partners	6.62 (5.61–7.81)	6.67 (5.65–7.87)	1.02 (0.79–1.31)	0.90	6.71 (5.22–8.59)	6.37 (4.92–8.20)	0.92 (0.62–1.36)	0.68
1 or more non-regular partner in the past year	19.3 (17.6–21.0)	20.2 (18.5–22.0)	1.11 (0.95–1.31)	0.20	14.8 (12.6–17.3)	13.4 (11.3–15.9)	1.11 (0.83–1.48)	0.48
Transactional sex in the past 1 month	7.39 (6.31–8.64)	6.78 (5.74–7.98)	0.95 (0.74–1.21)	0.66	5.67 (4.31–7.43)	6.94 (5.43–8.85)	1.31 (0.89–1.95)	0.17
STI symptoms in past 12 months	3.12 (2.44–3.97)	1.43 (0.99–2.06)	0.41 (0.26–0.65)	<0.001	2.20 (1.41–3.42)	1.04 (0.54–1.99)	0.51 (0.23–1.14)	0.10
Risk Category^‡^	**N = 3181**	**N = 2773**			**N = 1171**	**N = 1171**		
Not sexually active	30.4 (28.9–32.0)	29.2 (27.5–30.9)	0.96 (0.80–1.15)	0.63	28.8 (26.3–31.4)	20.7 (18.4–23.1)	0.70 (0.51–0.98)	0.04
Low risk	50.7 (48.9–52.5)	51.0 (49.1–52.9)	0.94 (0.83–1.08)	0.39	55.0 (52.1–57.8)	58.8 (55.9–61.5)	0.92 (0.74–1.13)	0.42
Medium risk	5.49 (4.70–6.41)	5.52 (4.73–6.43)	1.01 (0.80–1.28)	0.95	5.38 (4.22–6.83)	6.23 (4.98–7.77)	1.10 (0.77–1.57)	0.60
High risk	13.4 (12.3–14.7)	14.3 (13.0–15.6)	1.12 (0.96–1.31)	0.15	10.8 (9.19–12.8)	14.3 (12.5–16.5)	1.33 (1.03–1.71)	0.03
B) Females	**N = 4135**	**N = 3541**			**N = 1997**	**N = 1997**		
15-19yrs	**N = 1112**	**N = 734**			**N = 314**	**N = 314**		
Had sexual debut (15-19yrs)†	29.7 (27.1–32.4)	20.3 (17.5–23.4)	0.61 (0.49–0.77)	<0.001	23.9 (19.5–28.9)	33.8 (28.7–39.2)	1.64 (1.15–2.33)	0.01
15-54yrs started sex	**N = 3172**	**N = 2808**			**N = 1806**	**N = 1806**		
Multiple partners in past 1 month	1.06 (0.76–1.49)	0.89 (0.60–1.31)	0.82 (0.48–1.38)	0.46	0.94 (0.59–1.51)	0.89 (0.54–1.44)	0.93 (0.46–1.89)	0.84
Multiple partners in past 12 months	6.07 (5.29–6.95)	3.35 (2.74–4.08)	0.55 (0.43–0.72)	<0.001	4.87 (3.97–5.97)	2.44 (1.82–3.26)	0.51 (0.35–0.74)	<0.001
Concurrent partners	1.03 (0.73–1.45)	0.85 (0.57–1.27)	0.79 (0.46–1.36)	0.39	0.83 (0.50–1.37)	0.89 (0.54–1.44)	0.98 (0.48–2.03)	0.96
1 or more non-regular partner in the past year	8.30 (7.40–9.30)	7.34 (6.43–8.36)	0.99 (0.82–1.21)	0.96	6.31 (5.28–7.53)	3.88 (3.08–4.87)	0.67 (0.49–0.91)	0.01
Transactional Sex	6.90 (6.06–7.84)	5.48 (4.70–6.39)	0.81 (0.65–1.01)	0.06	6.81 (5.74–8.07)	4.60 (3.72–5.66)	0.69 (0.52–0.92)	0.01
Age disparate relationship 5 years or more	60.7 (58.9–62.5)	62.1 (60.3–63.9)	1.11 (0.99–1.24)	0.06	59.6 (57.3–61.9)	60.3 (57.9–62.5)	1.08 (0.94–1.24)	0.30
Age disparate relationship 10 years or more	22.1 (20.6–23.6)	21.6 (20.1–23.2)	0.97 (0.86–1.11)	0.69	21.6 (19.8–23.6)	20.6 (18.8–22.5)	0.93 (0.79–1.09)	0.37
STI symptoms in past 12 months	7.54 (6.66–8.53)	7.62 (6.70–8.66)	1.04 (0.85–1.26)	0.70	7.31 (6.19–8.60)	6.87 (5.79–8.13)	0.98 (0.76–1.27)	0.89
Risk Category^‡^	**N = 4135**	**N = 3541**			**N = 1997**	**N = 1997**		
Not sexually active	18.8 (17.8–20.0)	20.7 (19.4–22.1)	1.52 (1.29–1.79)	<0.001	14.1 (12.7–15.7)	12.1 (10.8–13.6)	1.72 (1.27–2.34)	0.001
Low risk	70.1 (68.7–71.5)	70.2 (68.7–71.7)	0.88 (0.78–1.00)	0.04	76.1 (74.1–77.9)	80.9 (79.1–82.5)	1.04 (0.86–1.26)	0.70
Medium risk	4.32 (3.72–5.01)	3.28 (2.74–3.92)	0.76 (0.59–0.97)	0.02	4.61 (3.77–5.62)	3.00 (2.34–3.85)	0.63 (0.45–0.87)	0.01
High risk	6.73 (6.00–7.55)	5.82 (5.09–6.64)	0.90 (0.75–1.09)	0.28	5.21 (4.31–6.27)	4.01 (3.23–4.96)	0.79 (0.58–1.07)	0.12

* In the cross-sectional analysis of the pre-covid survey, proportions are weighted to account for changes to selection between the surveys

† Odds ratios are adjusted for 5 year age group and site type. For variables limited to 15–19 year olds odds ratios are adjusted for site type only.

‡ Low risk = no risk behaviors, Medium risk = concurrent partners, more than one partner in the past 12 months, transactional sex, High risk = non-regular partners

For females, in the serial cross-sectional analysis, fewer of those aged 15–19 years reported having started sex during-Covid-19 than before Covid-19 (20.3% vs. 29.7%; AOR 0.49, 95% CI 0.38–0.63; Table 2). Amongst 15–54 year-old females who had started sex, fewer reported more than one sexual partner in the past 12 months during Covid-19 than before Covid-19 (3.35% vs. 6.07%; AOR 0.55, 95% CI 0.43–0.72) but no difference was found for multiple partners in the past month (p = 0.46). There was a trend towards less transactional sex in the past month (5.48% vs. 6.90%; AOR 0.81, 95% CI 0.65–1.01) which was statistically significant for females interviewed in the first period of the during-Covid-19 round (3.68% vs. 6.90%; AOR 0.55, 95% CI 0.34–0.90; S7 Table). No evidence was found for changes in the proportions of women with non-regular partners, concurrent partners or recent STI symptoms. In the cohort analysis, the proportion of females aged 15–19 years in the pre-Covid-19 survey round who had started sex increased from 23.9% pre-Covid-19 to 33.8% during-Covid-19. For females aged 15–54 years who had already started sex before Covid-19, after accounting for changes in age and site type, there were reductions in multiple sexual partners and non-regular partners in the past year, and in transactional sex (Table 2).

**Fig 3:**
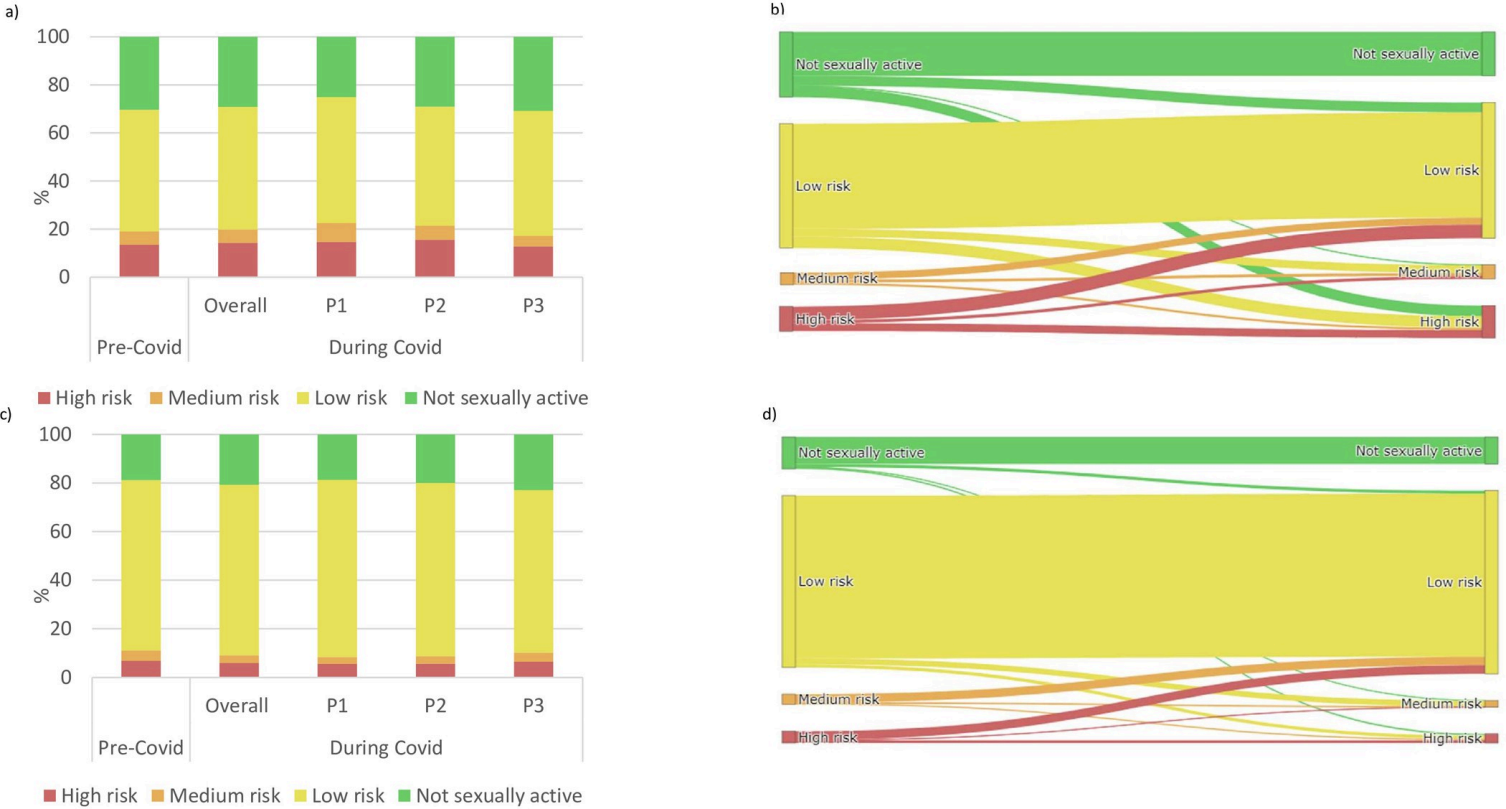
Changes in the proportion of adults 15–54 in each of the risk behaviour categories before and during Covid-19. a) Males–cross sectional analysis, b) Males in the cohort of individuals followed up, c) Females–cross sectional analysis, d) Females in the cohort of individuals.

Fig 3 compares the changes in the proportions of males and females in each of the summarised HIV risk categories from the pre-Covid-19 period to the during-Covid-19 period in the serial cross-sectional period data and in the cohort data. For males, in the period data, there were no changes in the aggregated proportions reporting the different levels of SRB (Fig 3A). However, the cohort analysis reveals extensive changes at the individual level with substantial proportions of previously low-risk men adopting SRBs during Covid-19 and *vice versa* (Fig 3B). For females, as noted above, the serial cross-sectional data show an increase during Covid-19 in the proportion not sexually-active. This is reflected here in the smaller proportions reporting low and medium (but not high) levels of SRB (Fig 3C). As for males, the female cohort analysis shows considerable movement at the individual level with a large fraction (75.0%) of those at high risk prior to Covid-19 adopting safer behaviours during Covid-19 but also a large fraction (37.5%) of those at high risk during Covid-19 being women who had previously been at low risk (Fig 3D).

## Discussion

We found evidence that large numbers of males and females in the general population who were HIV-negative at the onset of Covid-19 in Manicaland, east Zimbabwe, changed their sexual behaviour during the first year of the epidemic. In some cases, these individuals adopted behaviours that reduced their risk of acquiring HIV infection but, in other cases, individuals adopted more risky behaviours. As a consequence, for several indicators, the aggregate level of risk in the population remained unaltered. However, there was some evidence for reductions in population-level sexual risk behaviours during the worst of the Covid-19 epidemic. These included fewer young females during Covid-19 reporting recently starting sex and, for sexually-active females, reductions in reports of multiple sexual partners in the past 12 months and recent transactional sex. In the cohort analysis, fewer sexually-active females reported multiple sexual partners, non-regular partners and transactional sex. For males, the evidence for overall reductions in HIV risk was more limited but the proportion of sexually-active males interviewed in the first period of the during-Covid-19 survey round who reported multiple sexual partners in the past month was lower than in the pre-Covid-19 round.

We believe this is the first report on changes in SRBs during Covid-19 in a representative general population sample in a sub-Saharan African setting subject to a generalised HIV epidemic. However, studies of selected population sub-groups in Africa–e.g. women at high risk for HIV in Kenya [20] and antenatal clinic attendees in South Africa–have also found reductions in female SRB [19]. In Manicaland, these population-level reductions coincided with Government closures of beer-halls and restrictions on movements and social gatherings which all limited opportunities to meet partners. The restrictions on social gatherings–which included limits on wedding attendance (Fig 1)–together with the negative economic impact of the epidemic, probably explain the observed reductions in young females’ marriage and onset of sexual activity. For males, our finding of only a short-lived reduction in SRB early in the Covid-19 epidemic matches results from studies of MGDSM in high income countries [15–17].

Strengths of this study include the large representative general population sample and the availability of comparable data for the immediate pre-Covid-19 period and the period covering the first 12 months of the epidemic. The longitudinal cohort design permitted results from two different analytical approaches to be triangulated and allowed us to investigate changes at both the population level and the individual level. Limitations include the change in interview method from face-to-face interviews to telephone interviews which may have altered participation and social desirability biases in the data [27], particularly for sensitive topics like sexual behaviour. Whilst lower marriage rates may account for some of the reported reduction in young females starting sex, this reduction could be overestimated if more unmarried women started sex but did not report it due to the stigma surrounding women’s having sex before marriage [28, 29]. Changes observed in the cohort will reflect population ageing and other temporal factors not only the effects of Covid-19. Younger adults were over-sampled relative to older adults in the pre-Covid-19 survey but this was accounted for in the data analysis through use of weights and adjustment for age.

Mathematical model projections, done early in the Covid-19 pandemic, made what seemed at the time, to be a plausible, albeit speculative, assumption that SRB reductions would occur in both females and males and would offset any increases in HIV incidence due to interruptions in HIV prevention services [1]. In the Imperial College London model, when condom availability is disrupted for 6 months for 50% of the population, a 10% reduction in sexual contacts across all risk groups reduces a 12% increase in HIV incidence over a 1-year period (2020–2021) to a 6% decrease [1]. However, empirical data, such as that reported in this study, were lacking when these models were developed. Our findings of overall reductions in SRB in females but not in males during Covid-19 may have resulted in a more disassortative sexual mixing pattern in Manicaland–i.e. if the continuing high proportion of men with multiple or non-regular sexual partners during Covid-19 did so with a smaller group of higher-risk women–which, other things being equal, could have actually increased HIV risk within this population [10].

In this study, we identified a complex pattern of changing SRB during Covid-19 with HIV risk behaviours decreasing for some but increasing for others. Our results highlight that, even throughout periods of lockdown, when movement is restricted and social contacts are reduced, SRB remains high and HIV prevention programmes cannot be neglected despite the challenges of dealing with a pandemic. This has important policy implications, providing evidence for the continued provision of essential services for HIV prevention, such as pre-exposure prophylaxis and post-exposure prophylaxis, even during periods where social mixing is restricted. As the Covid-19 emergency eases, it will be important to further strengthen these programmes as more young women start sex and people who reduced their sexual activity during Covid-19 take on new partners which could lead to a surge in new infections.

## Supporting information

S1 Checklist Inclusivity in global research. (PDF)

S1 Fig Participation and eligibility flow diagram for the pre-Covid-19 survey round. (PDF)

S2 Fig Participation and eligibility flow diagram for the during Covid-19 survey round. (PDF)

S3 Fig Follow up flow diagram for cohort analysis. (PDF)

S1 Table Definitions of demographic characteristic variables. (PDF)

S2 Table Definitions of Covid-19 behaviours and responses variables. (PDF)

S3 Table Definitions of sexual risk behaviour variables. (PDF)

S4 Table Demographic characteristics of HIV negative males aged 15–54 in Manicaland, prior to and during three periods of the Covid-19 pandemic survey. February -March 2021; April ‐ May 2021; June ‐ July 2021.
(PDF)

S5 Table Demographic characteristics of HIV negative females aged 15–54 in Manicaland, prior to and during three periods of the Covid-19 pandemic survey. February -March 2021; April ‐ May 2021; June ‐ July 2021.
(PDF)

S6 Table Population effects and responses to the Covid-19 pandemic amongst HIV negative adults aged 15–54 in Manicaland during three periods of the Covid-19 pandemic survey. February -March 2021; April ‐ May 2021; June ‐ July 2021.
(PDF)

S7 Table Sexual risk behaviours of HIV negative adults aged 15–54 in Manicaland, prior to and during three periods of the Covid-19 pandemic survey. February -March 2021; April ‐ May 2021; June ‐ July 2021.
(PDF)

S1 Text Data source. Further detail regarding the source of data used in this analysis.
(PDF)

S2 Text Definitions of study measures. Supplementary detail regarding definitions of study measures including a) HIV status; b) Household wealth; c) Education; d) Risk categories.
(PDF)

S3 Text Periodic cross-sectional analysis. Explanation of analysis shown in S4–S7 Tables.
(PDF)
