# Correction: The impact of COVID-19 on sexual risk behaviour for HIV acquisition in east Zimbabwe: An observational study

**DOI:** 10.1371/journal.pgph.0004970

**Published:** 2025-07-22

**Authors:** Rebekah Morris, Simon Gregson, Rufurwokuda Maswera, Louisa Moorhouse, Tawanda Dadirai, Phyllis Mandizvidza, Brian Moyo, Owen Mugurungi, Constance Nyamukapa

There are errors in the Data Availability statement. The correct statement is: Due to the sensitive nature of data collected, including information on HIV status, treatment and sexual risk behaviour, the Manicaland Centre for Public Health Research does not make full analysis datasets publicly available. Summary datasets of household and background sociodemographic individual questionnaire data, covering rounds 1–8 (1998–2021), are publicly available for download via the Manicaland Centre for Public Health Research website here - http://www.manicalandhivproject.org/data-access.html. Quantitative data used for analyses produced by the Manicaland Centre for Public Health Research are available on request following completion of a data access request form here - http://www.manicalandhivproject.org/data-access.html. Additionally, summary HIV incidence and mortality data spanning rounds 1–6 (1998–2013), created in collaboration with the ALPHA Network are available via the DataFirst Repository here - https://www.datafirst.uct.ac.za/dataportal/index.php/catalog/ALPHA/about.

In Table 1, Section A for Males, Professional or Managerial under Employment, the p-value should be ‘0.002’ instead of ‘0.00.’. Please see the correct Table 1 here.

**Table 1:** Demographic characteristics of HIV negative adults aged 15-54 in Manicaland, prior to and during the Covid-19 pandemic.

	A) Males								B) Females							
	Cross-sectional analysis				Cohort analysis				Cross-sectional analysis				Cohort analysis			
	Pre-Covid	During Covid			Pre-Covid	During Covid			Pre-Covid	During Covid			Pre-Covid	During Covid		
	% (95%CI)^*^	% (95%CI)	AOR^†^ (95%CI)	p	% (95%CI)	% (95%CI)	AOR^†^ (95%CI)	p	% (95%CI)^*^	% (95%CI)	AOR^†^ (95%CI)	p	% (95%CI)	% (95%CI)	AOR^†^ (95%CI)	p
**15-54yrs**	**N = 3181**	**N = 2773**			**N = 1171**	**N = 1171**			**N = 4135**	**N = 3541**			**N = 1997**	**N = 1997**		
**Age Group**																
15-19	26.4 (25.0-27.9)	25.8 (24.2-27.5)			25.8 (23.4-28.4)	17.8 (15.7-20.1)			21.5 (20.4-22.7)	20.7 (19.4-22.1)			15.7 (14.2-17.4)	10.6 (9.34-12.0)		
20-24	17.6 (16.4-18.9)	16.1 (14.8-17.5)			12.7 (10.9-14.8)	15.5 (13.6-17.7)			18.8 (17.7-19.9)	15.9 (14.7-17.1)			14.6 (13.1-16.2)	12.8 (11.4-14.3)		
25-29	11.7 (10.7-12.8)	12.3 (11.1-13.5)			9.74 (8.16-11.6)	10.8 (9.19-12.8)			13.8 (12.7-15.0)	14.5 (13.4-15.7)			13.8 (12.3-15.4)	15.2 (13.7-16.8)		
30-34	12.3 (11.1-13.7)	10.4 (9.34-11.6)			11.5 (9.82-13.5)	10.6 (8.95-12.5)			13.7 (12.6-14.9)	12.5 (11.4-13.6)			15.7 (14.1-17.3)	13.9 (12.5-15.5)		
35-44	20.2 (18.7-21.8)	21.8 (20.3-23.4)			24.4 (22.0-27.0)	25.7 (23.3-28.3)			20.2 (18.9-21.5)	22.7 (21.3-24.1)			25.2 (23.3-27.1)	27.6 (25.7-29.6)		
45-54	11.8 (10.6-13.1)	13.6 (12.3-14.9)			15.8 (13.8-18.0)	16.5 (14.5-18.7)			12.0 (10.9-13.1)	13.7 (12.6-14.9)			15.0 (13.5-16.7)	16.4 (14.9-18.1)		
**55+**						3.07 (2.22-4.23)								3.51 (2.78-4.41)		
**Site Type** ^‡^																
Urban	14.5 (13.3-15.7)	20.6 (19.1-22.1)	1.53 (1.33-1.75)	<0.001	14.8 (12.9-16.9)	14.8 (12.9-16.9)	0.99 (0.78-1.25)	0.93	18.7 (17.6-20.0)	19.5 (18.2-20.8)	1.06 (0.95-1.19)	0.32	15.6 (14.0-17.2)	15.6 (14.0-17.2)	1.04 (0.87-1.23)	0.69
Periurban	23.1 (21.6-24.6)	27.8 (26.1-29.5)	1.28 (1.14-1.44)	<0.001	25.7 (23.3-28.3)	25.7 (23.3-28.3)	1.03 (0.85-1.24)	0.75	28.1 (26.7-29.5)	30.3 (28.8-31.9)	1.12 (1.01-1.24)	0.03	30.2 (28.3-32.3)	30.2 (28.3-32.3)	1.01 (0.88-1.16)	0.84
Estates	32.6 (30.9-34.3)	24.8 (23.3-26.5)	0.68 (0.61-0.77)	<0.001	30.3 (27.7-33.0)	30.3 (27.7-33.0)	1.00 (0.83-1.20)	0.99	21.9 (20.7-23.3)	21.2 (19.9-22.6)	0.95 (0.85-1.06)	0.34	21.1 (19.4-23.0)	21.1 (19.4-23.0)	0.98 (0.84-1.14)	0.78
Rural	29.9 (28.3-31.5)	26.8 (25.2-28.5)	0.86 (0.76-0.96)	0.01	29.2 (26.7-31.9)	29.2 (26.7-31.9)	0.98 (0.82-1.17)	0.81	31.2 (29.8-32.7)	29.0 (27.5-30.5)	0.89 (0.81-0.99)	0.03	33.0 (31.0-35.1)	33.0 (31.0-35.1)	0.98 (0.86-1.12)	0.79
**Marital Status**																
Never married	43.7 (42.0-45.5)	43.3 (41.5-45.2)	1.08 (0.91-1.29)	0.36	38.4 (35.7-41.3)	33.7 (31.1-36.5)	1.13 (0.83-1.55)	0.44	23.8 (22.6-25.1)	23.9 (22.5-25.3)	1.17 (1.02-1.35)	0.03	17.7 (16.1-19.4)	15.2 (13.7-16.9)	1.34 (1.05-1.71)	0.02
Married/cohabiting	52.5 (50.8-54.3)	53.4 (51.5-55.3)	0.97 (0.83-1.14)	0.69	59.1 (56.2-61.9)	62.9 (60.0-65.6)	0.83 (0.63-1.10)	0.20	64.8 (63.3-66.2)	63.0 (61.4-64.6)	0.85 (0.76-0.95)	0.003	71.6 (69.6-73.5)	73.1 (71.1-75.0)	0.92 (0.78-1.08)	0.30
Divorced/Separated	3.51 (2.88-4.28)	2.92 (2.36-3.62)	0.86 (0.63-1.17)	0.34	2.22 (1.52-3.24)	3.07 (2.22-4.23)	1.29 (0.77-2.17)	0.33	8.58 (7.73-9.52)	9.86 (8.92-10.9)	1.14 (0.97-1.33)	0.12	7.66 (6.57-8.91)	7.41 (6.34-8.65)	0.88 (0.69-1.13)	0.32
Widowed	0.23 (0.10-0.51)	0.36 (0.19-0.67)	1.35 (0.48-3.78)	0.57	0.26 (0.08-0.79)	0.34 (0.13-0.91)	1.30 (0.29-5.85)	0.73	2.80 (2.30-3.40)	3.28 (2.74-3.92)	1.10 (0.82-1.46)	0.53	3.05 (2.38-3.91)	4.26 (3.45-5.24)	1.04 (0.72-1.51)	0.83
**Household Wealth Index** ^§^																
Poorest	9.81 (8.79-10.9)	5.59 (4.80-6.51)	0.55 (0.45-0.68)	<0.001	9.65 (8.08-11.5)	7.34 (5.98-8.99)	0.72 (0.53-0.99)	0.04	8.97 (8.12-9.90)	6.70 (5.92-7.57)	0.74 (0.62-0.88)	0.001	9.51 (8.30-10.9)	8.17 (7.04-9.45)	0.84 (0.67-1.06)	0.15
2nd poorest	46.5 (44.8-48.3)	42.1 (40.3-44.0)	0.98 (0.88-1.10)	0.77	42.7 (39.9-45.6)	42.5 (39.7-45.4)	1.00 (0.84-1.19)	0.99	40.3 (38.8-41.9)	41.4 (39.8-43.1)	1.11 (1.00-1.22)	0.04	41.5 (39.4-43.7)	42.9 (40.7-45.1)	1.06 (0.93-1.22)	0.38
3rd poorest	22.3 (20.8-23.8)	29.3 (27.7-31.1)	1.41 (1.25-1.59)	<0.001	24.0 (21.6-26.5)	28.4 (25.9-31.1)	1.26 (1.04-1.52)	0.02	24.0 (22.7-25.4)	28.1 (26.7-29.6)	1.22 (1.10-1.36)	<0.001	24.3 (22.5-26.2)	26.2 (24.3-28.1)	1.11 (0.96-1.28)	0.17
4th poorest	20.1 (18.8-21.6)	22.2 (20.7-23.8)	0.90 (0.78-1.03)	0.12	22.1 (19.8-24.6)	20.9 (18.7-23.3)	0.91 (0.73-1.13)	0.40	25.0 (23.7-26.3)	22.7 (21.4-24.1)	0.81 (0.72-0.91)	<0.001	23.3 (21.5-25.2)	21.8 (20.0-23.7)	0.89 (0.76-1.05)	0.18
Least poor	1.25 (0.91-1.70)	0.76 (0.49-1.16)	0.52 (0.30-0.89)	0.02	1.54 (0.97-2.43)	0.77 (0.40-1.47)	0.91 (0.73-1.13)	0.40	1.70 (1.34-2.15)	1.02 (0.73-1.41)	0.58 (0.39-0.88)	0.01	1.40 (0.97-2.02)	1.00 (0.65-1.55)	0.74 (0.41-1.32)	0.31
**Employment**																
Professional or managerial	4.03 (3.35-4.85)	6.31 (5.46-7.28)	1.48 (1.15-1.91)	0.002	4.95 (3.85-6.36)	6.40 (5.14-7.96)	1.20 (0.83-1.73)	0.33	3.63 (3.06-4.30)	4.69 (4.04-5.44)	1.21 (0.95-1.54)	0.12	3.61 (2.87-4.52)	4.61 (3.77-5.62)	1.20 (0.87-1.66)	0.27
Self-employed: small business	1.17 (0.83-1.63)	1.33 (0.97-1.84)	0.96 (0.60-1.55)	0.87	1.20 (0.71-2.01)	1.28 (0.77-2.11)	1.03 (0.49-2.15)	0.95	0.46 (0.29-0.76)	1.47 (1.12-1.92)	3.36 (1.90-5.93)	<0.001	0.60 (0.34-1.06)	1.60 (1.14-2.26)	2.48 (1.26-4.88)	0.01
Skilled labour	7.40 (6.48-8.43)	14.3 (13.1-15.7)	2.42 (2.01-2.92)	<0.001	8.28 (6.83-10.0)	15.8 (13.8-18.0)	1.96 (1.49-2.58)	<0.001	2.34 (1.90-2.89)	4.52 (3.88-5.25)	1.97 (1.50-2.58)	<0.001	2.40 (1.82-3.18)	3.76 (3.00-4.69)	1.47 (1.01-2.15)	0.04
Manual/unskilled labour	18.8 (17.5-20.3)	15.5 (14.2-16.9)	0.99 (0.84-1.16)	0.86	13.8 (12.0-15.9)	17.6 (15.5-19.9)	1.32 (1.03-1.69)	0.03	4.42 (3.80-5.13)	7.79 (6.96-8.72)	1.90 (1.55-2.34)	<0.001	4.36 (3.54-5.35)	8.21 (7.09-9.50)	2.00 (1.51-2.65)	<0.001
Informal employment	15.0 (13.7-16.3)	14.1 (12.8-15.4)	0.81 (0.69-0.94)	0.01	17.0 (14.9-19.3)	16.0 (14.0-18.2)	0.84 (0.67-1.06)	0.14	13.7 (12.6-14.8)	18.8 (17.6-20.1)	1.41 (1.24-1.60)	<0.001	15.9 (14.4-17.6)	23.6 (21.8-25.6)	1.56 (1.33-1.84)	<0.001
Student	22.2 (20.8-23.6)	19.8 (18.4-21.4)	0.77 (0.64-0.92)	0.004	24.2 (21.8-26.7)	11.7 (10.0-13.7)	0.32 (0.23-0.45)	<0.001	15.2 (14.3-16.3)	15.3 (14.2-16.6)	1.13 (0.96-1.32)	0.15	12.7 (11.3-14.2)	8.06 (6.95-9.34)	0.78 (0.58-1.04)	0.09
Unemployed	24.8 (23.3-26.4)	28.6 (27.0-30.3)	1.20 (1.06-1.35)	0.004	23.7 (21.3-26.2)	31.3 (28.7-34.0)	1.44 (1.18-1.74)	<0.001	52.5 (51.0-54.1)	47.4 (45.7-49.0)	0.82 (0.75-0.90)	<0.001	52.8 (50.6-55.0)	50.1 (47.9-52.3)	0.84 (0.73-0.95)	0.01
Other: unspecified^‖^	6.61 (5.76-7.57)				6.92 (5.60-8.52)				7.64 (6.84-8.53)				7.61 (6.53-8.86)			
**Alcohol/Drugs**																
Drank alcohol in the past year	26.9 (25.3-28.5)	21.7 (20.2-23.2)	0.73 (0.64-0.84)	<0.001	27.3 (24.8-30.0)	26.5 (24.0-29.1)	0.86 (0.71-1.05)	0.13	2.01 (1.62-2.50)	2.40 (1.94-2.96)	1.19 (0.87-1.62)	0.27	2.10 (1.56-2.83)	1.85 (1.35-2.55)	0.84 (0.53-1.33)	0.46
Visited bar, beerhall or shebeen in past month	31.0 (29.3-32.7)	16.9 (15.6-18.4)	0.44 (0.39-0.51)	<0.001	31.4 (28.8-34.1)	21.4 (19.1-23.9)	0.52 (0.43-0.63)	<0.001	0.62 (0.42-0.91)	0.62 (0.41-0.94)	0.97 (0.55-1.71)	0.91	0.40 (0.20-0.80)	0.60 (0.34-1.06)	1.42 (0.57-3.55)	0.45
Using drugs for pleasure	4.78 (4.08-5.61)	2.92 (2.36-3.62)	0.60 (0.45-0.79)	<0.001	3.76 (2.81-5.01)	3.67 (2.73-4.92)	0.90 (0.58-1.39)	0.63	0.19 (0.10-0.39)	0.34 (0.19-0.60)	1.81 (0.73-4.48)	0.20	0.05 (0.01-0.35)	0.30 (0.13-0.67)	6.50 (0.78-54.27)	0.08
**15-19yrs**	**N = 986**	**N = 716**			**N = 302**	**N = 302**			**N = 1112**	**N = 734**			**N = 314**	**N = 314**		
**Currently Enrolled in Education**	73.2 (70.4-75.9)	64.0 (60.4-67.4)	0.64 (0.50-0.82)	<0.001	84.4 (79.9-88.1)	37.7 (32.4-43.4)	0.33 (0.21-0.52)	<0.001	57.7 (54.8-60.6)	57.1 (53.5-60.6)	1.06 (0.84-1.33)	0.64	69.4 (64.1-74.3)	36.0 (30.8-41.5)	0.90 (0.59-1.37)	0.62

* In the cross-sectional analysis of the pre-covid survey, proportions are weighted to account for age bias in selection.

† Odds ratios are adjusted for 5 year age group and site type. For variables limited to 15–19 year olds odds ratios are adjusted for site type and age in years.

‡ Note that no specific procedures were followed to identify individuals moving to different study site between the survey rounds.

§ Household wealth was estimated for each individual by assessing asset ownership within their household and attributing a score as described by Schur et al [19]. Each asset variable was transformed into scores between 0 and 1. The values of each asset variable were then summed and divided by the total number of assets giving an overall score between 0 and 1. Equally spaced cut offs (0,0.2, 0.4, 0.6, 0.8) were used to categorise the overall wealth of a household into five groups.

‖ These individuals answered’other’ for their category of employment but then did not specify in the follow up question so they could not be recoded to any of the other categories.

In Fig 2, there is a minor error in the placement of an asterisk and the relevant footnote is missing. Please see the correct Fig 2 here.

**Fig 2:**
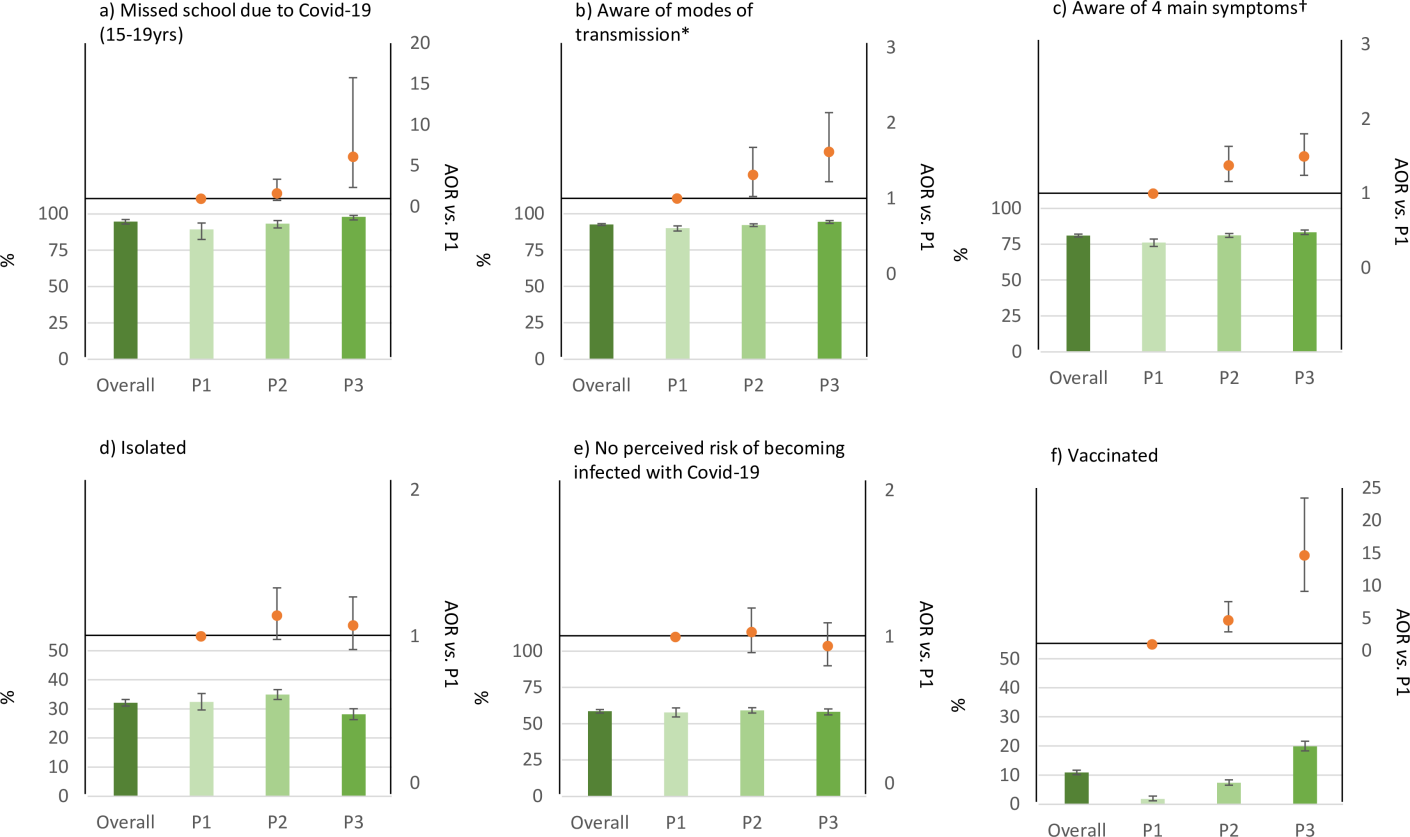
Population effects and responses to the Covid-19 pandemic amongst HIV negative adults aged 15–54 in Manicaland during three periods of the Covid-19 pandemic from February to July 2020 (P1: February-March 2020, P2: April-May 2020, P3: June-July 2020). * Modes of transmission included: droplets from coughing or sneezing, touching eyes/ears/nose or mouth when unclean and touching surfaces. † Fever, cough, loss of smell, and loss of sense of taste.
