# Supplementary material for: The impact of COVID-19 on sexual risk behaviour for HIV acquisition in east Zimbabwe: An observational study
Source: PLOS Glob Public Health. 2024 Jul 17;4(7):e0003194. doi: 10.1371/journal.pgph.0003194 (PMC11253984; doi:10.1371/journal.pgph.0003194)
Supplement: S1 Checklist — (PDF) [file pgph.0003194.s001.pdf]

# Inclusivity in global research

PLOS' policy on inclusivity in global research aims to improve transparency in the reporting of research performed outside of researchers' own country or community and ensures that PLOS publications reporting global research adhere to high standards for research ethics and authorship. Authors of relevant research articles may be asked to complete the questionnaire below, which outlines ethical, cultural, and scientific considerations specific to inclusivity in global research. This questionnaire may be requested when researchers have travelled to a different country to conduct research, if research uses samples collected in another country, research with Indigenous populations or their lands, or if research is on cultural artefacts. Researchers travelling to another country solely to use laboratory equipment will not normally be required to complete the questionnaire. However, the questionnaire can be requested at the journal's discretion for any submission – if you have been requested to complete this questionnaire by the PLOS journal you submitted to, please do so.

Please complete the questionnaire below and include this as a Supporting Information file with your manuscript. Note that if your paper is accepted for publication, this checklist will be published with your article in the supporting information files. Please ensure that you reference the checklist in the main body of your manuscript. We suggest adding a subsection 'Inclusivity in global research' to your Methods section and adding the following sentence: "Additional information regarding the ethical, cultural, and scientific considerations specific to inclusivity in global research is included in the Supporting Information (SX Checklist)"

The questions have been designed to be applicable to a wide range of study types, and there are subsections for both human subjects research and non-human subjects research. If any of the questions are not relevant to your research please mark them as "N/A" as appropriate.

## Ethical considerations, permits and authorship

*This section is applicable to all research types.*

Provide details as to who granted permissions and/or consent for the study to take place in the Methods section of your manuscript. This should include the names of **all** ethics boards, governmental organizations, community leaders or other bodies that provided approval for the study. If individuals provided approval refer to these people by their role or title but do not list their name(s).

| |
|---|
| Reported on page number: 6 |

If there were any deviations from the study protocol after approval was obtained please provide details of these changes in the Methods section of your manuscript.

| |
|---|
| Reported on page number: NA |

Did this study involve local collaborators that are residents of the country where the research was conducted or members of the community studied? If you do not have any authors from said communities, please provide an explanation for this below.

Yes. This study involved local collaborators from the Manicaland Centre for Public Health which is part of the Biomedical Research and Training Institute of Zimbabwe and from the Zimbabwe Ministry of Health and Child Care Zimbabwe.

Everyone listed as an author should meet PLOS' criteria for authorship and all individuals who meet these criteria should be included in the author byline, rather than the acknowledgements. For further information please see the journal's Authorship Policy.

## Human subjects research (e.g. health research, medical research, cross-cultural psychology)

Did you obtain written informed consent from a representative of the local community or region before the research took place? How did you establish who speaks for the community? Details of written informed consent obtained from study participants should be reported separately in the Methods section of your manuscript.

Written approval for the study was obtained from the Research Council of Zimbabwe, which has overall statutory responsibility for all research conducted in the country, from the Permanent Secretary in the Ministry of Health and Child Care (MoHCC), and from the Medical Research Council of Zimbabwe (MRCZ). Verbal informed consent for the research was also obtained from local community leaders (Chiefs and Village Headmen / Kraal Heads) within the study areas. The Manicaland Centre team has been carrying out research with the study populations for more than 25 years and has good knowledge of the local communities and its leadership structures.

Details of the MRCZ-approved informed consent process for individual research participants within the study communities that was applied during Covid-19 are reported separately in the Methods section of the manuscript.

How did members of the local community provide input on the aims of the research investigation, its methodology, and its anticipated outcome(s)?

The study protocol was developed in partnership with local Zimbabwean partners. Dr Constance Nyamukapa (Biomedical Research and Training Institute [BRTI]) was a principal investigator and Dr Owen Mugurungi (Zimbabwe Ministry of Health and Child Care) was a co-investigator on the initial study protocol. One of the study investigators (Prof Gregson) led the Zimbabwe MoHCC Covid-19 Research Consortium's Surveillance and Epidemiology Working Group which identified priorities for research on the local population impact of the Covid-19 pandemic.

When engaging with the local community, how did you ensure that the informed consent documents and other materials could be understood by local stakeholders?

Informed consent documents and other materials for the study were developed using MRCZ templates and guidelines produced locally to be understandable by local stakeholders. The resulting documents were then reviewed by local BRTI IRB and MRCZ reviewers and approved as suitable for use by local stakeholders by their respective committees.

Research assistants, with Good Clinical Practice certificates and trained on how to appropriately inform potential participants about the study and to request informed consent, contacted eligible study participants. Informed consent was sought on a one-to-one basis after ensuring that the respondent was in a private space. Research assistants read out the consent form to the potential participant over the phone in the local language (Shona) or in English depending on the respondent's preference.

Will the findings of the research be made available in an understandable format to stakeholders in the community where the study was conducted (e.g. via a presentation, summary report, copies of publications, etc.)? Please provide details of how this will be achieved.

Key findings from the research were summarised in interim reports (http://www.manicalandhivproject.org/covid-19-hiv-risk-survey-reports.html) and submitted monthly to the Chief Coordinator of the Covid-19 Response in the Zimbabwe President's Office and to the Director of the MoHCC's AIDS and TB Department. A Zoom meeting was held with the MoHCC's AIDS and TB Department at which the research results were presented. At the end of the study, summary reports (translated into Shona – the local language) were produced for each of the 8 study communities and presented and discussed at a series of local community meetings (http://www.manicalandhivproject.org/hiv-epidemic-status-reports-2018-2023.html).

## Non-human subjects research using specimens/ animals collected as part of the study, or those housed in archival collections. Examples include archaeology, paleontology, botany and zoology.

Did the permission you obtained from a local authority to perform the study include an agreement on access to outputs and benefit sharing? This may include procedures to enable fair distribution of the benefits and resources arising from the research performed. Please include any details of Prior Informed Consent and

NA

Benefit Sharing Agreements obtained. These may be required by field-specific regulations, for example the Convention on Biological Diversity (CBD) and the associated Nagoya Protocol.

If the material used in your study was imported, please A) provide the year it was imported and B) indicate whether permits were obtained to import/export the materials used, C) provide details of any permits obtained. If this information is not available, please indicate this.

NA

If you used archival specimens, please state how the material used in your study was acquired by the institute it is held in and provide details of any permits obtained for the original excavations/ sample collection. If this information is not available, please indicate this.

NA

How was the potential cultural significance of the materials collected in your study to local communities considered in your research design? Were Indigenous peoples and/or local researchers and institutions involved with archaeological excavations / collection of specimens? If so, please provide a description of their involvement.

NA

If your manuscript includes photographs of human remains please indicate whether authors obtained permission from descendants or affiliated cultural communities to do so.

NA
