# Supplementary material for: The impact of COVID-19 on sexual risk behaviour for HIV acquisition in east Zimbabwe: An observational study
Source: PLOS Glob Public Health. 2024 Jul 17;4(7):e0003194. doi: 10.1371/journal.pgph.0003194 (PMC11253984; doi:10.1371/journal.pgph.0003194)
Supplement: S2 Fig — (PDF) [file pgph.0003194.s003.pdf]

S2 Fig. Participation and Eligibility flow diagram for the during Covid-19 survey round.

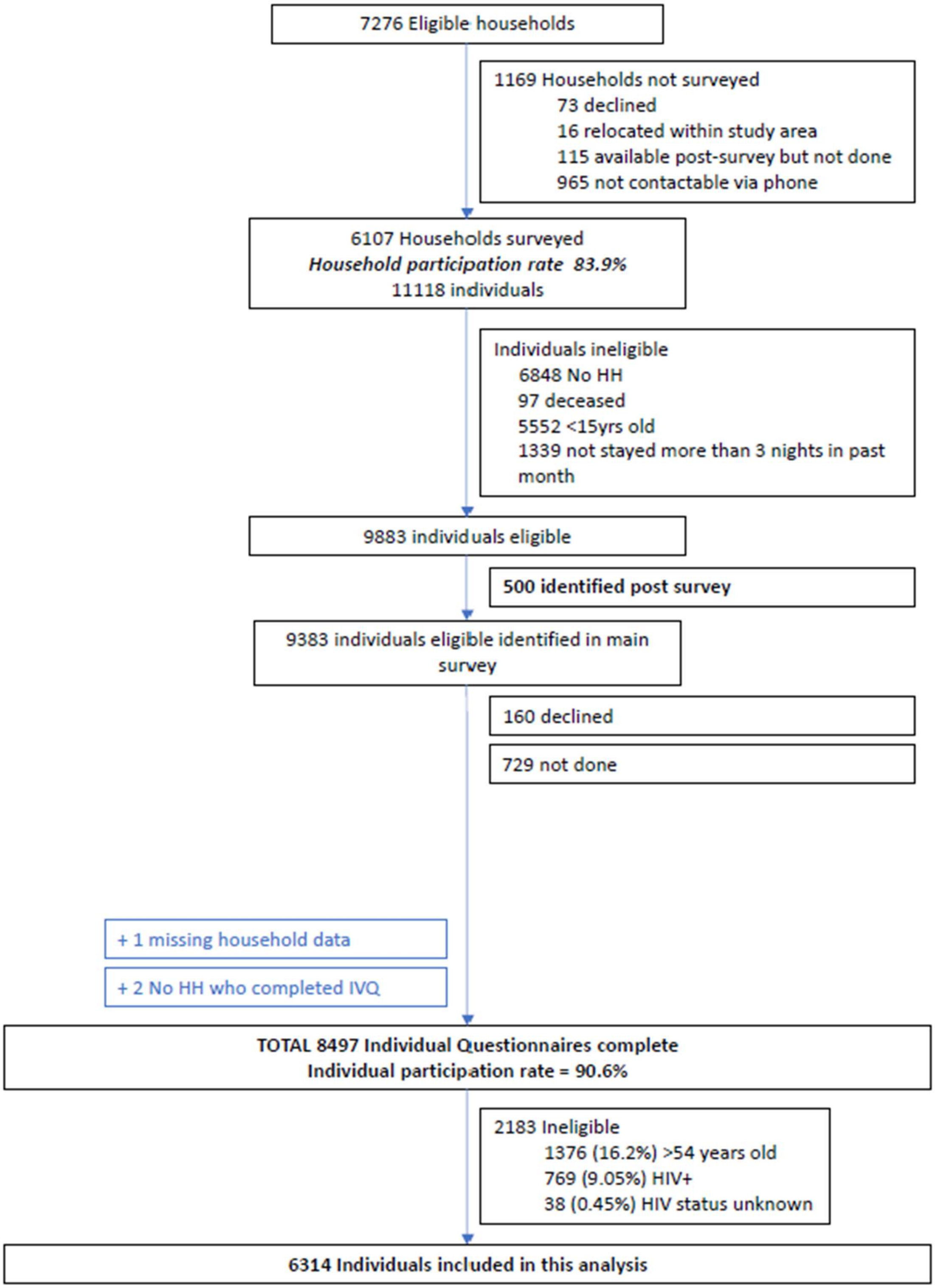

HHQ = household questionnaire
IVQ = Individual questionnaire
