# Supplementary material for: The impact of COVID-19 on sexual risk behaviour for HIV acquisition in east Zimbabwe: An observational study
Source: PLOS Glob Public Health. 2024 Jul 17;4(7):e0003194. doi: 10.1371/journal.pgph.0003194 (PMC11253984; doi:10.1371/journal.pgph.0003194)
Supplement: S3 Fig — (PDF) [file pgph.0003194.s004.pdf]

S3 Fig. Follow up flow diagram for cohort analysis.

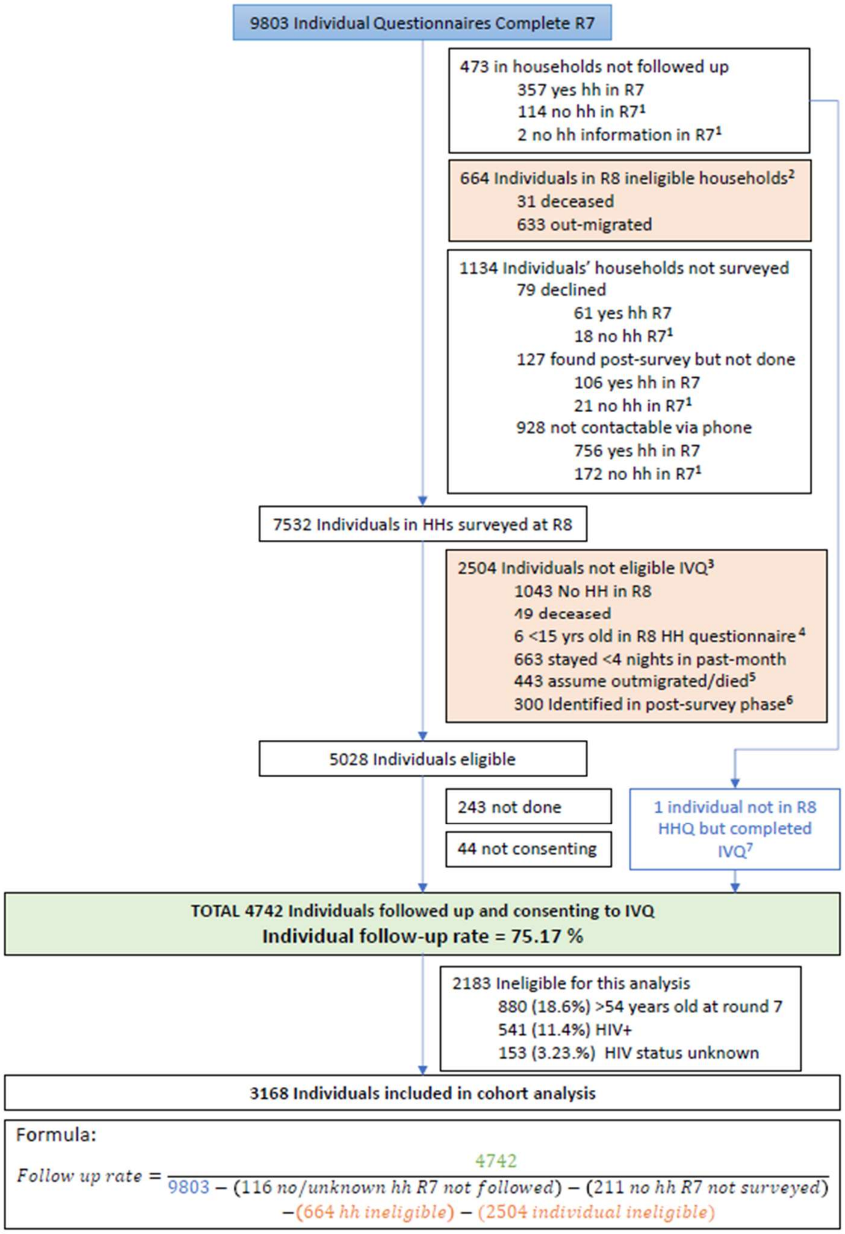

Notes:

1. Participants from 'No household' in R7 should have also been 'No household' at R8 if their household was followed up. These individuals are therefore excluded from denominator for calculation of individual follow-up rates as they would have been ineligible for IVQ.
2. These R7 participants were in ineligible households in R8
3. These R7 participants were ineligible at R8 at an individual level even though their household was surveyed
4. 6 individuals reported their age to be greater than 15 in R7 IVQ, however, in R8 their age is reported as <15 yrs in the household survey making them ineligible for IVQ in R8
5. 443 R7 participants' households were followed up in R8 but they were not listed as household members – we assume these either left the household or died.
6. 300 otherwise eligible individuals were from households which were not identified in the main R8 survey so therefore were not eligible for IVQ.
7. 1 R7 participant was followed up to R8 IVQ despite their household not being followed up in R8. They were from a yes household in R7.
