# Supplementary material for: The impact of COVID-19 on sexual risk behaviour for HIV acquisition in east Zimbabwe: An observational study
Source: PLOS Glob Public Health. 2024 Jul 17;4(7):e0003194. doi: 10.1371/journal.pgph.0003194 (PMC11253984; doi:10.1371/journal.pgph.0003194)
Supplement: S1 Table — (PDF) [file pgph.0003194.s005.pdf]

S1 Table. Definitions of demographic characteristic variables.

	Survey Question(s)	*Notes*
**HIV status**	See 2.1	
**Marital Status**	Q: Have you ever been MARRIED or in a long-term or COHABITING relationship? * Q: Are you currently widowed, divorced or separated from your most recent spouse/partner?<br>• *Widowed*<br>• *Divorced*<br>• *Separated*<br>• *Still in union*	Relationships of 12months or more were considered "long term"
**Household Wealth index**	See 2.2	
**Highest Level of Education Achieved**	Q: How old were you when you left school?<br>• never been to school = 99<br>Q: What is the highest grade of school you have completed?<br>• None<br>• Primary<br>• Secondary<br>• Higher	
**Employment**	Q: In which sector of employment do you work?<br>• Estates: tea, coffee, forestry etc<br>• Manufacturing or building trade<br>• Police or army<br>• Teacher: primary school or secondary school<br>• Nurse or doctor<br>• Services or retail shops<br>• Informal: petty trading (veg etc)<br>• Informal: subsistence agriculture<br>• Student<br>• Unemployed: excl. agriculture<br>• Office worker<br>• Other (specify)<br>Q: What type of work do you do?<br>• Professional or managerial<br>• Self-employed: small business<br>• Skilled labour<br>• Manual / unskilled labour	
**Currently enrolled in Education**	Q: Are you currently ENROLLED in school full-time?	
**Drank alcohol in the past year**	Q: Over the last 12 months, how many times per month, on average, have you had a drink containing alcohol?	
**Visited bar, beerhall or shebeen in the past month**	Q: How many times have you visited a bar or beerhall or shebeen in the last month?	
**Using drugs for pleasure**	Q: Do you take any of these types of drugs for pleasure?<br>a) Injecting drugs<br>b) Drugs you smoke<br>c) Prescription Drugs<br>d) Other drugs you swallow	
