# Supplementary material for: The impact of COVID-19 on sexual risk behaviour for HIV acquisition in east Zimbabwe: An observational study
Source: PLOS Glob Public Health. 2024 Jul 17;4(7):e0003194. doi: 10.1371/journal.pgph.0003194 (PMC11253984; doi:10.1371/journal.pgph.0003194)
Supplement: S2 Table — (PDF) [file pgph.0003194.s006.pdf]

S2 Table. Definitions of Covid-19 behaviours and responses variables.

	Survey Question(s)	*Notes*
**Missed School due to Covid-19**	Q: How many times in the last 12 months have you been unable to attend school for COVID-19-related reasons?	Answers of 1 or more were coded as 'yes' for this variable.
**Aware of 4 main symptoms of Covid-19**	Q; What are the main symptoms of the new coronavirus, COVID-19?<br>**a) Fever**<br>**b) Cough -dry or productive**<br>c) Tiredness<br>d) Muscle/joint pain<br>e) Shortness of breath<br>f) Runny or blocked nose<br>g) Sore throat<br>**h) Loss of smell**<br>**i) Loss of sense of taste**<br>j) Loss of appetite<br>k) Diarhoea<br>l) Headache<br>m) Vomiting<br>n) Don't know of any<br>o) Other (specify)	Responses including all of 'Fever', 'Cough', 'Loss of smell', and 'Loss of sense of taste' were coded as 'yes' for this variable.
**Aware of main modes of transmission**	Q: What are the ways in which the new corona virus (COVID-19) can be spread from person to person)<br>a) Droplets from coughing and sneezing<br>b) Touching other people who have the virus<br>c) Touching surfaces which have the virus on<br>d) Touching your eyes, nose, or mouth with unclean hands<br>e) Body fluids from an infected person<br>f) Faeces of an infected<br>g) Don't know of any<br>h) Others (specify)	Respondents who answered yes to all three of; 'Droplets from coughing or sneezing', 'touching eyes/ears/nose or mouth when unclean' and 'touching surfaces were coded as 'yes'.
**Relocated**	Q: Have you taken any of the following steps to shield or self -isolate yourself from becoming infected with the COVID-19 virus at any time since April 2020?<br>*a) Working from home*<br>***b) Relocating to rural home***<br>*c) self-isolated at home*<br>*d) other (specify)*<br>*e) NA -under 50 & no health condition*	Respondents who answered yes to 'Relocating to rural home' were coded as having relocated
**Isolated**	Q: Have you taken any of the following steps to shield or self -isolate yourself from becoming infected with the COVID-19 virus at any time since April 2020?<br>***f) Working from home***<br>*g) Relocating to rural home*<br>***h) Self-isolated at home***<br>*i) other (specify)*<br>*NA -under 50 & no health condition*	Respondents who answered yes to 'Working from home' or 'Self-isolated at home' were coded as having isolated.
**Wore a facemask outside**	Q: In the LAST 2 WEEKS, how many times did you?<br>a) Wear a face mask outside:<br>• Most of the times<br>• Some of the times<br>• Seldom<br>• Never	

**Ever tested for COVID-19**	Q: On how many different occasions have you been tested for infection with the COVID-19 virus?<br>• never tested = 99	
**No perceived chance of becoming infected with Covid in next 12 months**	Q: What are the chances you will get infected with the COVID-19 virus in the next 12 months?<br>• Certain /almost certain<br>• High<br>• Moderate<br>• Small<br>• None	Those answering 'None' were coded as 'no perceived risk'
**Vaccinated against COVID-19**	Q: Have you received the COVID-19 vaccine yourself?<br>• YES<br>• NO<br>• Don't Know<br>• Covid-19 vaccine not available yet	
