# Supplementary material for: The impact of COVID-19 on sexual risk behaviour for HIV acquisition in east Zimbabwe: An observational study
Source: PLOS Glob Public Health. 2024 Jul 17;4(7):e0003194. doi: 10.1371/journal.pgph.0003194 (PMC11253984; doi:10.1371/journal.pgph.0003194)
Supplement: S3 Table — (PDF) [file pgph.0003194.s007.pdf]

S3 Table. Definitions of Sexual Risk Behaviour variables.

	During-Covid Survey Questions Used	*Notes*
Had sexual debut	Q: How old were you when you had sex for the first time?<br>• Not yet had sex = 99	
Multiple partners in past 1 month	Q: How many different partners have you had sex within the LAST MONTH?	
Multiple partners in past 12 months	Q: How many different sexual partners have you had in the LAST 12 MONTHS?*	If the sum of regular and non-regular partners did not add to the total number of partners in the past 12 months respondents were asked this question again.
Concurrent partners	Q: How many sexual relationships do you consider yourself to be involved in at the moment?<br>- ≥2	
1 or more non-regular partner in the past year	Q: How many of the sexual partners that you had in the last 12 months were NON-REGULAR partners?<br>- ≥1	
Transactional sex in the past 1 month	Q4: Have you given or received money, goods or services in exchange for sex with this person in the last month?<br>- Asked for each of their three most recent sexual partners	
STI symptoms in past 12 months	Q: For men: Some men experience white, transparent or yellowish discharge from the penis, which might not cause discomfort or may be accompanied by mild burning paid on urination. In the last 12 months, have you had these symptoms?<br>- 1 - Yes- discharge & pain (both)<br>- 2 - Yes - discharge only<br>- 3 - Yes - pain only<br>- 4 - No<br>- 98 - Don't know<br>Q: Some (wo)men experience sores in the genital area. During the last 12 months, have you noticed any such sores?<br>- 1 - Yes- discharge & pain (both)<br>- 2 - Yes - discharge only<br>- 3 - Yes - pain only<br>- 4 - No<br>- 98 - Don't know	Options 1, 2 or 3 were considered a positive response.
