# Supplementary material for: The impact of COVID-19 on sexual risk behaviour for HIV acquisition in east Zimbabwe: An observational study
Source: PLOS Glob Public Health. 2024 Jul 17;4(7):e0003194. doi: 10.1371/journal.pgph.0003194 (PMC11253984; doi:10.1371/journal.pgph.0003194)
Supplement: S4 Table — February -March 2021; April ‐ May 2021; June ‐ July 2021. (PDF) [file pgph.0003194.s008.pdf]

S4 Table. Demographic characteristics of HIV negative males aged 15-54 in Manicaland, prior to and during three periods of the Covid-19 pandemic survey.

	Pre-Covid-19	During Covid-19 - Period 1			During Covid-19 - Period 2			During Covid-19 - Period 3		
	% (95%CI)*	% (95%CI)	AOR† (95%CI)	p	% (95%CI)	AOR† (95%CI)	p	% (95%CI)	AOR† (95%CI)	p
**A) Males**	**N=3181**	**N=418**			**N=1233**			**N=1122**		
**Age Group**										
15-19	26.4 (25.0 - 27.9)	25.1 (21.2 - 29.5)	-	-	25.1 (22.7 - 27.6)	-	-	26.9 (24.4 - 29.6)	-	-
20-24	17.6 (16.4 - 18.9)	14.1 (11.1 - 17.8)	-	-	17.3 (15.3 - 19.5)	-	-	15.6 (13.6 - 17.8)	-	-
25-29	11.7 (10.7 - 12.8)	12.2 (9.39 - 15.7)	-	-	11.7 (10.0 - 13.6)	-	-	12.9 (11.1 - 15.0)	-	-
30-34	12.3 (11.1 - 13.7)	10.8 (8.13 - 14.1)	-	-	10.5 (8.95 - 12.4)	-	-	10.2 (8.52 - 12.1)	-	-
35-44	20.2 (18.7 - 21.8)	21.5 (17.8 - 25.7)	-	-	21.7 (19.4 - 24.0)	-	-	22.1 (19.8 - 24.6)	-	-
45-54	11.8 (10.6 - 13.1)	16.3 (13.0 - 20.1)	-	-	13.8 (12.0 - 15.8)	-	-	12.3 (10.5 - 14.4)	-	-
**Site type**										
Urban	14.5 (13.3 - 15.7)	25.6 (21.6 - 30.0)	2.07 (1.62 - 2.63)	<0.001	29.9 (27.4 - 32.5)	2.54 (2.17 - 2.97)	<0.001	8.47 (6.97 - 10.2)	0.54 (0.43 - 0.68)	<0.001
Periurban	23.1 (21.6 - 24.6)	23.2 (19.4 - 27.5)	1.00 (0.79 - 1.28)	0.97	22.0 (19.8 - 24.4)	0.94 (0.80 - 1.10)	0.45	35.8 (33.1 - 38.7)	1.86 (1.61 - 2.16)	<0.001
Estates	32.6 (30.9 - 34.3)	29.9 (25.7 - 34.5)	0.88 (0.70 - 1.10)	0.27	28.4 (25.9 - 31.0)	0.82 (0.71 - 0.95)	0.01	19.1 (16.9 - 21.5)	0.49 (0.41 - 0.58)	<0.001
Rural	29.9 (28.3 - 31.5)	21.3 (17.6 - 25.5)	0.63 (0.49 - 0.81)	<0.001	19.7 (17.6 - 22.0)	0.57 (0.49 - 0.67)	<0.001	36.6 (33.9 - 39.5)	1.35 (1.17 - 1.56)	<0.001
**Marital Status**										
Never Married	43.7 (42.0 - 45.5)	39.2 (34.7 - 44.0)	0.87 (0.61 - 1.25)	0.47	43.8 (41.0 - 46.6)	1.15 (0.91 - 1.44)	0.23	44.3 (41.4 - 47.2)	1.10 (0.87 - 1.39)	0.43
Married/cohabiting	52.5 (50.8 - 54.3)	57.2 (52.4 - 61.9)	1.14 (0.82 - 1.57)	0.44	52.3 (49.5 - 55.1)	0.88 (0.71 - 1.08)	0.21	53.2 (50.3 - 56.1)	1.02 (0.82 - 1.26)	0.88
Divorced/seperated	3.51 (2.88 - 4.28)	2.87 (1.63 - 4.99)	0.81 (0.43 - 1.49)	0.49	3.41 (2.53 - 4.58)	0.99 (0.67 - 1.45)	0.95	2.41 (1.65 - 3.49)	0.74 (0.48 - 1.15)	0.18
Widowed	0.23 (0.10 - 0.51)	0.72 (0.23 - 2.21)	2.64 (0.64 - 10.81)	0.18	0.49 (0.22 - 1.08)	1.95 (0.61 - 6.24)	0.26	0.09 (0.01 - 0.63)	0.32 (0.04 - 2.68)	0.29
**Household Wealth Index**										
Poorest	9.81 (8.79 - 10.9)	11.0 (8.34 - 14.4)	1.47 (1.03 - 2.08)	0.03	6.17 (4.96 - 7.66)	0.80 (0.61 - 1.05)	0.11	2.94 (2.10 - 4.11)	0.21 (0.15 - 0.31)	<0.001
2nd Poorest	46.5 (44.8 - 48.3)	46.7 (41.9 - 51.5)	1.31 (1.05 - 1.65)	0.02	38.0 (35.3 - 40.8)	0.93 (0.80 - 1.08)	0.34	44.9 (42.0 - 47.8)	0.94 (0.81 - 1.09)	0.42
3rd Poorest	22.3 (20.8 - 23.8)	34.0 (29.6 - 38.7)	1.66 (1.33 - 2.08)	<0.001	30.2 (27.7 - 32.8)	1.36 (1.17 - 1.58)	<0.001	26.6 (24.1 - 29.3)	1.37 (1.17 - 1.61)	<0.001
4th Poorest	20.1 (18.8 - 21.6)	8.37 (6.07 - 11.4)	0.22 (0.15 - 0.31)	<0.001	24.5 (22.1 - 26.9)	0.82 (0.69 - 0.98)	0.03	24.9 (22.4 - 27.5)	1.45 (1.21 - 1.74)	<0.001
Least Poor	1.25 (0.91 - 1.70)	0	-	-	1.14 (0.67 - 1.91)	0.80 (0.43 - 1.50)	0.49	0.62 (0.30 - 1.30)	0.41 (0.18 - 0.93)	0.03
**Highest Level of Education Acheived¶**										
None/Primary	10.4 (9.31 - 11.5)	16.5 (13.2 - 20.4)	1.90 (1.42 - 2.54)	<0.001	15.2 (13.3 - 17.4)	1.78 (1.46 - 2.17)	<0.001	14.3 (12.3 - 16.4)	1.49 (1.21 - 1.84)	<0.001
Secondary/Higher	89.6 (88.5 - 90.7)	83.5 (79.6 - 86.8)	0.53 (0.39 - 0.70)	<0.001	84.8 (82.6 - 86.7)	0.56 (0.46 - 0.69)	<0.001	85.7 (83.6 - 87.7)	0.67 (0.54 - 0.83)	<0.001
**Employment**										
Professional or managerial	4.03 (3.35 - 4.85)	4.31 (2.73 - 6.74)	0.93 (0.55 - 1.56)	0.78	6.41 (5.17 - 7.92)	1.52 (1.11 - 2.07)	0.01	6.95 (5.60 - 8.60)	1.67 (1.22 - 2.29)	0.001
Self-employed: small business	1.17 (0.83 - 1.63)	0.48 (0.12 - 1.90)	0.34 (0.08 - 1.42)	0.14	1.14 (0.67 - 1.91)	0.86 (0.45 - 1.64)	0.64	1.87 (1.22 - 2.85)	1.27 (0.73 - 2.21)	0.40
Skilled labour	7.40 (6.48 - 8.43)	9.33 (6.88 - 12.5)	1.30 (0.89 - 1.88)	0.17	15.1 (13.2 - 17.2)	2.36 (1.88 - 2.95)	<0.001	15.3 (13.3 - 17.6)	3.09 (2.45 - 3.90)	<0.001
Manual / unskilled labour	18.8 (17.5 - 20.3)	8.37 (6.07 - 11.4)	0.36 (0.24 - 0.53)	<0.001	16.8 (14.8 - 19.0)	0.99 (0.80 - 1.21)	0.89	16.8 (14.7 - 19.1)	1.39 (1.12 - 1.72)	0.003
Informal employment	15.0 (13.7 - 16.3)	21.5 (17.8 - 25.7)	1.46 (1.11 - 1.92)	0.01	12.0 (10.3 - 13.9)	0.68 (0.55 - 0.84)	<0.001	13.5 (11.7 - 15.7)	0.74 (0.60 - 0.92)	0.01
Student	22.2 (20.8 - 23.6)	17.5 (14.1 - 21.4)	0.62 (0.42 - 0.90)	0.01	18.5 (16.4 - 20.8)	0.67 (0.53 - 0.85)	0.001	22.2 (19.9 - 24.7)	0.96 (0.76 - 1.23)	0.77
Unemployed	24.8 (23.3 - 26.4)	38.5 (34.0 - 43.3)	2.21 (1.76 - 2.78)	<0.001	30.1 (27.6 - 32.7)	1.42 (1.21 - 1.66)	<0.001	23.4 (21.0 - 25.9)	0.79 (0.67 - 0.93)	0.01
Other: unspecified‖	6.61 (5.76 - 7.57)	-	-	-	-	-	-	-	-	-
**Currently Enrolled in Education**	24.3 (22.9 - 25.7)	16.5 (13.2 - 20.4)	0.44 (0.31 - 0.64)	0.000	17.44 (15.42 - 19.66)	0.48 (0.38 - 0.60)	0.000	21.1 (18.8 - 23.6)	0.70 (0.55 - 0.88)	0.002
**Alcohol/Drugs**										
Drank Alcohol in the past year	26.9 (25.3 - 28.5)	27.3 (23.2 - 31.8)	1.02 (0.80 - 1.30)	0.89	23.5 (21.2 - 26.0)	0.84 (0.71 - 1.00)	0.05	17.6 (15.4 - 19.9)	0.54 (0.45 - 0.65)	<0.001
Visited Bar, beerhall or shebeen in past month	31.0 (29.3 - 32.7)	19.9 (16.3 - 24.0)	0.55 (0.43 - 0.72)	<0.001	18.0 (16.0 - 20.3)	0.51 (0.43 - 0.60)	<0.001	14.6 (12.7 - 16.8)	0.35 (0.29 - 0.42)	<0.001
Using drugs for pleasure	4.78 (4.08 - 5.61)	4.07 (2.54 - 6.45)	0.85 (0.50 - 1.42)	0.53	2.84 (2.04 - 3.93)	0.58 (0.39 - 0.84)	0.005	2.58 (1.80 - 3.70)	0.53 (0.35 - 0.79)	0.002
**Males 15-19**	**N=986**	**N=105**			**N=309**			**N=302**		
**Currently Enrolled in Education**	73.2 (70.4 - 75.9)	61.9 (52.2 - 70.8)	0.51 (0.31 - 0.84)	0.008	58.58 (52.98 - 63.96)	0.46 (0.34 - 0.64)	0.63	70.2 (64.8 - 75.1)	1.71 (0.94 - 3.10)	0.08

* In the cross-sectional analysis of the pre-covid survey, proportions are weighted to account for age bias in selection

† Odds ratios are adjusted for 5 year age group and site type. For variables limited to 15-19 year olds odds ratios are adjusted for site type only.

§Household wealth was estimated for each individual by assessing asset ownership within their household and attributing a score as described by Schur et al.19 Each asset variable was transformed into scores between 0 and 1. The values of each asset variable were then summed and divided by the total number of assets giving an overall score between 0 and 1. Equally spaced cut offs (0,0.2, 0.4, 0.6, 0.8) were used to categorise the overall wealth of a household into five groups.

¶ Education variable inconsistently coded in during covid survey - for cohort individuals (in both analyses), education was updated based on answer pre-covid if level of education was higher in R7.

|| These individuals answered 'other' for their category of employment but then did not specify in the follow up question so they could not be recoded to any of the other categories.
