# Supplementary material for: The impact of COVID-19 on sexual risk behaviour for HIV acquisition in east Zimbabwe: An observational study
Source: PLOS Glob Public Health. 2024 Jul 17;4(7):e0003194. doi: 10.1371/journal.pgph.0003194 (PMC11253984; doi:10.1371/journal.pgph.0003194)
Supplement: S5 Table — February -March 2021; April ‐ May 2021; June ‐ July 2021. (PDF) [file pgph.0003194.s009.pdf]

S5 Table. Demographic characteristics of HIV negative females aged 15-54 in Manicaland, prior to and during three periods of the Covid-19 pandemic survey.

	Pre-Covid-19	During Covid-19 - Period 1			During Covid-19 - Period 2			During Covid-19 - Period 3		
	% (95%CI)*	% (95%CI)	AOR† (95%CI)	p	% (95%CI)	AOR† (95%CI)	p	% (95%CI)	AOR† (95%CI)	p
**B) Females**	**N=4135**	**N=635**			**N=1785**			**N=1121**		
**Age Group**										
15-19	21.5 (20.4 - 22.7)	19.7 (16.8 - 23.0)			19.7 (17.9 - 21.6)			23.0 (20.6 - 25.6)		
20-24	18.8 (17.7 - 19.9)	15.3 (12.7 - 18.3)			15.5 (13.9 - 17.3)			16.8 (14.7 - 19.1)		
25-29	13.8 (12.7 - 15.0)	15.4 (12.8 - 18.5)			14.5 (12.9 - 16.2)			14.1 (12.2 - 16.3)		
30-34	13.7 (12.6 - 14.9)	11.3 (9.09 - 14.1)			12.3 (10.9 - 13.9)			13.4 (11.5 - 15.5)		
35-44	20.2 (18.9 - 21.5)	22.7 (19.6 - 26.1)			23.6 (21.7 - 25.7)			21.1 (18.8 - 23.6)		
45-54	12.0 (10.9 - 13.1)	15.6 (13.0 - 18.6)			14.4 (12.8 - 16.1)			11.6 (9.85 - 13.6)		
**Site type**										
Urban	18.7 (17.6 - 20.0)	27.6 (24.2 - 31.2)	1.69 (1.40 - 2.05)	<0.001	23.0 (21.1 - 25.0)	1.32 (1.15 - 1.51)	<0.001	9.37 (7.79 - 11.2)	0.45 (0.36 - 0.55)	<0.001
Periurban	28.1 (26.7 - 29.5)	29.4 (26.0 - 33.1)	1.08 (0.89 - 1.29)	0.44	21.6 (19.7 - 23.5)	0.70 (0.62 - 0.81)	<0.001	44.8 (41.9 - 47.7)	2.08 (1.81 - 2.38)	<0.001
Estates	21.9 (20.7 - 23.3)	23.5 (20.3 - 26.9)	1.08 (0.88 - 1.31)	0.48	24.7 (22.8 - 26.8)	1.15 (1.01 - 1.32)	0.03	14.4 (12.4 - 16.5)	0.60 (0.50 - 0.72)	<0.001
Rural	31.2 (29.8 - 32.7)	19.5 (16.6 - 22.8)	0.53 (0.43 - 0.65)	<0.001	30.8 (28.7 - 32.9)	0.97 (0.86 - 1.10)	0.63	31.5 (28.8 - 34.3)	1.01 (0.88 - 1.17)	0.84
**Marital Status**										
Never Married	23.8 (22.6 - 25.1)	23.6 (20.5 - 27.1)	1.27 (0.97 - 1.68)	0.09	23.0 (21.1 - 25.0)	1.21 (1.01 - 1.46)	0.04	25.4 (23.0 - 28.1)	1.07 (0.86 - 1.32)	0.54
Married/cohabiting	64.8 (63.3 - 66.2)	65.2 (61.4 - 68.8)	0.94 (0.77 - 1.16)	0.57	63.2 (60.9 - 65.4)	0.81 (0.71 - 0.92)	0.002	61.5 (58.6 - 64.3)	0.86 (0.73 - 1.01)	0.07
Divorced/seperated	8.58 (7.73 - 9.52)	7.56 (5.74 - 9.89)	0.80 (0.58 - 1.10)	0.17	10.6 (9.24 - 12.1)	1.22 (1.01 - 1.48)	0.04	10.0 (8.37 - 11.9)	1.21 (0.96 - 1.52)	0.11
Widowed	2.80 (2.30 - 3.40)	3.62 (2.42 - 5.40)	1.16 (0.72 - 1.89)	0.54	3.25 (2.52 - 4.18)	1.03 (0.73 - 1.45)	0.87	3.12 (2.25 - 4.32)	1.18 (0.78 - 1.78)	0.43
**Household Wealth Index§**										
Poorest	8.97 (8.12 - 9.90)	10.6 (8.39 - 13.2)	1.65 (1.23 - 2.21)	0.001	6.96 (5.87 - 8.24)	0.81 (0.65 - 1.01)	0.07	4.11 (3.09 - 5.44)	0.36 (0.26 - 0.49)	<0.001
2nd Poorest	40.3 (38.8 - 41.9)	48.5 (44.6 - 52.4)	1.86 (1.54 - 2.23)	<0.001	39.9 (37.6 - 42.2)	1.01 (0.90 - 1.15)	0.83	39.9 (37.1 - 42.8)	0.96 (0.84 - 1.11)	0.61
3rd Poorest	24.0 (22.7 - 25.4)	31.0 (27.5 - 34.7)	1.31 (1.09 - 1.58)	0.004	28.3 (26.2 - 30.4)	1.19 (1.05 - 1.35)	0.01	26.3 (23.8 - 28.9)	1.22 (1.05 - 1.43)	0.01
4th Poorest	25.0 (23.7 - 26.3)	9.92 (7.82 - 12.5)	0.22 (0.16 - 0.29)	<0.001	23.6 (21.7 - 25.6)	0.86 (0.75 - 1.00)	0.05	28.6 (26.0 - 31.3)	1.28 (1.09 - 1.51)	0.003
Least Poor	1.70 (1.34 - 2.15)	0	-	-	1.29 (0.86 - 1.94)	0.82 (0.50 - 1.32)	0.41	1.16 (0.67 - 1.99)	0.59 (0.32 - 1.08)	0.09
**Highest Level of Education Acheived¶**										
None/Primary	16.9 (15.8 - 18.1)	18.7 (15.9 - 22.0)	1.11 (0.89 - 1.39)	0.36	17.1 (15.4 - 18.9)	0.99 (0.85 - 1.15)	0.85	18.8 (16.6 - 21.2)	1.25 (1.05 - 1.49)	0.01
Secondary/Higher	83.1 (81.9 - 84.2)	81.3 (78.0 - 84.1)	0.90 (0.72 - 1.12)	0.36	82.9 (81.1 - 84.6)	1.01 (0.87 - 1.18)	0.85	81.2 (78.8 - 83.4)	0.80 (0.67 - 0.96)	0.01
**Employment**										
Professional or managerial	3.63 (3.06 - 4.30)	2.52 (1.55 - 4.08)	0.59 (0.35 - 1.01)	0.05	4.26 (3.41 - 5.30)	1.10 (0.82 - 1.48)	0.52	6.60 (5.29 - 8.21)	1.77 (1.30 - 2.41)	<0.001
Self-employed: small business	0.46 (0.29 - 0.76)	2.20 (1.31 - 3.69)	4.47 (2.14 - 9.33)	<0.001	1.51 (1.04 - 2.20)	3.24 (1.73 - 6.09)	<0.001	0.98 (0.54 - 1.76)	2.75 (1.25 - 6.04)	0.01
Skilled labour	2.34 (1.90 - 2.89)	2.36 (1.43 - 3.88)	0.92 (0.52 - 1.61)	0.77	4.59 (3.71 - 5.67)	1.86 (1.36 - 2.55)	<0.001	5.62 (4.41 - 7.13)	2.98 (2.11 - 4.21)	<0.001
Manual / unskilled labour	4.42 (3.80 - 5.13)	5.20 (3.72 - 7.22)	1.12 (0.75 - 1.67)	0.57	9.41 (8.14 - 10.9)	2.16 (1.71 - 2.72)	<0.001	6.69 (5.37 - 8.31)	1.98 (1.48 - 2.67)	<0.001
Informal employment	13.7 (12.6 - 14.8)	25.2 (22.0 - 28.7)	2.03 (1.64 - 2.50)	<0.001	17.8 (16.1 - 19.7)	1.31 (1.12 - 1.53)	0.001	16.8 (14.7 - 19.1)	1.24 (1.03 - 1.50)	0.02
Student	15.2 (14.3 - 16.3)	13.9 (11.4 - 16.8)	1.03 (0.74 - 1.42)	0.87	15.2 (13.6 - 17.0)	1.24 (1.00 - 1.52)	0.05	16.3 (14.3 - 18.6)	1.03 (0.81 - 1.31)	0.79
Unemployed	52.5 (51.0 - 54.1)	48.7 (44.8 - 52.6)	0.89 (0.75 - 1.06)	0.19	47.2 (44.9 - 49.5)	0.81 (0.72 - 0.91)	<0.001	47.0 (44.1 - 49.9)	0.81 (0.71 - 0.93)	0.003
Other: unspecified‖	7.64 (6.84 - 8.53)									
**Alcohol/Drugs**										
Drank Alcohol in the past year	2.01 (1.62 - 2.50)	1.26 (0.63 - 2.50)	0.56 (0.27 - 1.17)	0.12	2.46 (1.84 - 3.30)	1.23 (0.85 - 1.80)	0.27	2.94 (2.10 - 4.11)	1.53 (1.01 - 2.33)	0.05
Visited Bar, beerhall or shebeen in past month	0.62 (0.42 - 0.91)	0.31 (0.08 - 1.25)	0.50 (0.12 - 2.11)	0.34	0.50 (0.26 - 0.97)	0.89 (0.42 - 1.92)	0.77	0.98 (0.54 - 1.76)	1.27 (0.62 - 2.60)	0.51
Using drugs for pleasure	0.19 (0.10 - 0.39)	0.16 (0.02 - 1.11)	0.86 (0.11 - 7.00)	0.89	0.39 (0.19 - 0.82)	2.13 (0.76 - 5.95)	0.15	0.36 (0.13 - 0.95)	1.82 (0.54 - 6.17)	0.34
**Females 15-19**	**N=1112**	**N=125**			**N=351**			**N=258**		
**Currently Enrolled in Education**	57.7 (54.8 - 60.6)	45.6 (37.0 - 54.5)	0.52 (0.33 - 0.83)	0.006	55.56 (50.30 - 60.69)	0.96 (0.72 - 1.29)	0.78	64.7 (58.7 - 70.3)	1.70 (1.21 - 2.40)	0.002

* In the cross-sectional analysis of the pre-covid survey, proportions are weighted to account for age bias in selection

† Odds ratios are adjusted for 5 year age group and site type. For variables limited to 15-19 year olds odds ratios are adjusted for site type only.

§Household wealth was estimated for each individual by assessing asset ownership within their household and attributing a score as described by Schur et al.19 Each asset variable was transformed into scores between 0 and 1. The values of each asset variable were then summed and divided by the total number of assets giving an overall score between 0 and 1. Equally spaced cut offs (0,0.2, 0.4, 0.6, 0.8) were used to categorise the overall wealth of a household into five groups.

¶ Education variable inconsistently coded in during covid survey - for cohort individuals (in both analyses), education was updated based on answer pre-covid if level of education was higher in R7.

‖ These individuals answered 'other' for their category of employment but then did not specify in the follow up question so they could not be recoded to any of the other categories.
