# Supplementary material for: The impact of COVID-19 on sexual risk behaviour for HIV acquisition in east Zimbabwe: An observational study
Source: PLOS Glob Public Health. 2024 Jul 17;4(7):e0003194. doi: 10.1371/journal.pgph.0003194 (PMC11253984; doi:10.1371/journal.pgph.0003194)
Supplement: S6 Table — February -March 2021; April ‐ May 2021; June ‐ July 2021. (PDF) [file pgph.0003194.s010.pdf]

S6 Table. Population effects and responses to the Covid-19 pandemic amongst HIV negative adults aged 15-54 in Manicaland during three periods of the Covid-19 pandemic survey from February to July 2021.

	Overall	Period 1	Period 2			Period 3		
	% (95%CI)	% (95%CI)	% (95%CI)	AOR (95%CI)*	p value	% (95%CI)	AOR (95%CI)*	p value
**A) Males**								
**15-19yrs enrolled in education**	**N=716**	**N=105**	**N=309**			**N=302**		
Missed School due to Covid-19	94.3 (91.8 - 96.1)	84.6 (73.5 - 91.6)	93.4 (88.7 - 96.2)	2.40 (0.97 - 5.91)	0.06	98.1 (95.1 - 99.3)	8.85 (2.53 - 30.9)	0.001
**15-54yrs**	**N=2773**	**N=418**	**N=1233**			**N=1122**		
Aware of 4 main symptoms of Covid-19	75.8 (74.2 - 77.4)	71.5 (67.0 - 75.7)	73.7 (71.2 - 76.1)	1.12 (0.87 - 1.44)	0.37	79.8 (77.3 - 82.0)	1.36 (1.05 - 1.78)	0.02
Aware of main modes of transmission	88.4 (87.1 - 89.5)	80.6 (76.5 - 84.1)	87.3 (85.4 - 89.1)	1.70 (1.25 - 2.30)	0.001	92.3 (90.6 - 93.8)	2.40 (1.70 - 3.38)	<0.001
Relocated	5.70 (4.89 - 6.62)	9.57 (7.09 - 12.8)	6.00 (4.80 - 7.47)	0.58 (0.38 - 0.87)	0.01	3.92 (2.93 - 5.23)	0.45 (0.29 - 0.72)	0.001
Isolated	21.0 (19.5 - 22.5)	30.9 (26.6 - 35.5)	22.1 (19.8 - 24.5)	0.60 (0.47 - 0.78)	<0.001	16.0 (14.0 - 18.3)	0.55 (0.42 - 0.72)	<0.001
Wore a facemask outside	96.6 (95.9 - 97.2)	96.4 (94.1 - 97.8)	96.4 (95.1 - 97.3)	0.96 (0.53 - 1.75)	0.90	97.0 (95.8 - 97.8)	1.13 (0.60 - 2.14)	0.70
Ever tested for COVID-19	9.20 (8.17 - 10.3)	9.09 (6.68 - 12.3)	8.76 (7.30 - 10.5)	0.89 (0.60 - 1.32)	0.56	9.71 (8.11 - 11.6)	1.41 (0.94 - 2.12)	0.09
No perceived chance of becoming infected with Covid in next 12 months	60.9 (59.0 - 62.8)	63.0 (58.1 - 67.6)	59.3 (56.5 - 62.1)	0.83 (0.65 - 1.06)	0.13	61.9 (58.9 - 64.7)	0.84 (0.66 - 1.08)	0.18
Vaccinated against COVID-19	10.5 (9.37 - 11.7)	1.91 (0.96 - 3.79)	7.46 (6.12 - 9.07)	4.16 (1.99 - 8.66)	<0.001	16.9 (14.8 - 19.2)	11.3 (5.47 - 23.3)	<0.001
**B) Females**								
**15-19yrs enrolled in education**	**N=734**	**N=125**	**N=351**			**N=258**		
Missed School due to Covid-19	95.2 (92.7 - 96.9)	94.7 (84.6 - 98.3)	93.3 (88.8 - 96.1)	0.82 (0.22 - 3.06)	0.77	97.6 (93.8 - 99.1)	2.62 (0.55 - 12.4)	0.22
**15-54yrs**	**N=3541**	**N=635**	**N=1785**			**N=1121**		
Aware of 4 main symptoms of Covid-19	85.3 (84.1 - 86.4)	79.2 (75.9 - 82.2)	86.3 (84.6 - 87.8)	1.74 (1.37 - 2.21)	<0.001	87.1 (85.0 - 88.9)	1.89 (1.45 - 2.47)	<0.001
Aware of main modes of transmission	96.0 (95.3 - 96.6)	96.2 (94.4 - 97.5)	95.5 (94.5 - 96.4)	0.86 (0.54 - 1.37)	0.51	96.5 (95.3 - 97.4)	1.11 (0.66 - 1.89)	0.69
Relocated	3.61 (3.05 - 4.28)	2.36 (1.43 - 3.88)	5.10 (4.17 - 6.22)	2.40 (1.37 - 4.22)	0.002	1.96 (1.30 - 2.96)	1.40 (0.71 - 2.77)	0.33
Isolated	40.8 (39.2 - 42.5)	33.4 (29.8 - 37.2)	43.8 (41.5 - 46.1)	1.71 (1.40 - 2.09)	<0.001	40.3 (37.5 - 43.2)	1.96 (1.57 - 2.44)	<0.001
Wore a facemask outside	98.4 (97.9 - 98.7)	95.7 (93.9 - 97.1)	98.8 (98.2 - 99.2)	3.52 (1.95 - 6.34)	<0.001	99.1 (98.3 - 99.5)	6.78 (3.22 - 14.3)	<0.001
Ever tested for COVID-19	11.1 (10.1 - 12.1)	9.13 (7.12 - 11.6)	9.08 (7.83 - 10.5)	1.05 (0.76 - 1.44)	0.77	15.3 (13.3 - 17.6)	1.93 (1.40 - 2.67)	<0.001
No perceived chance of becoming infected with Covid in next 12 months	56.8 (55.1 - 58.4)	54.4 (50.5 - 58.4)	59.1 (56.7 - 61.4)	1.22 (1.01 - 1.48)	0.04	54.3 (51.3 - 57.3)	0.93 (0.75 - 1.14)	0.47
Vaccinated against COVID-19	11.3 (10.3 - 12.4)	1.73 (0.96 - 3.10)	7.39 (6.27 - 8.71)	5.10 (2.73 - 9.54)	<0.001	22.9 (20.6 - 25.5)	17.9 (9.66 - 33.3)	<0.001
**C) All adults**								
**15-19yrs**	**N=1450**	**N=230**	**N=660**			**N=560**		
Missed School due to Covid-19	94.8 (93.1 - 96.1)	89.3 (82.4 - 93.7)	93.4 (90.3 - 95.5)	1.63 (0.80 - 3.34)	0.18	97.9 (95.8 - 98.9)	5.88 (2.31 - 15.0)	<0.001
**15-54yrs**	**N=6314**	**N=1053**	**N=3018**			**N=2243**		
Aware of 4 main symptoms of Covid-19	81.1 (80.1 - 82.1)	76.2 (73.5 - 78.6)	81.1 (79.7 - 82.5)	1.38 (1.16 - 1.63)	<0.001	83.4 (81.8 - 84.9)	1.50 (1.24 - 1.80)	<0.001
Aware of main modes of transmission	92.6 (91.9 - 93.2)	90.0 (88.1 - 91.7)	92.2 (91.2 - 93.1)	1.31 (1.03 - 1.68)	0.03	94.4 (93.4 - 95.3)	1.62 (1.23 - 2.14)	0.001
Relocated	4.53 (4.04 - 5.07)	5.22 (4.03 - 6.74)	5.47 (4.71 - 6.34)	1.04 (0.76 - 1.43)	0.79	2.94 (2.32 - 3.73)	0.75 (0.52 - 1.10)	0.14
Isolated	32.1 (31.0 - 33.3)	32.4 (29.6 - 35.3)	34.9 (33.2 - 36.6)	1.14 (0.98 - 1.33)	0.09	28.2 (26.4 - 30.1)	1.08 (0.91 - 1.27)	0.39
Wore a facemask outside	97.6 (97.2 - 97.9)	96.0 (94.6 - 97.0)	97.8 (97.2 - 98.3)	1.84 (1.24 - 2.74)	0.002	98.0 (97.4 - 98.5)	2.25 (1.45 - 3.50)	<0.001
Ever tested for COVID-19	10.2 (9.52 - 11.0)	9.12 (7.52 - 11.0)	8.95 (7.98 - 10.0)	1.00 (0.78 - 1.28)	0.99	12.5 (11.2 - 14.0)	1.67 (1.29 - 2.14)	<0.001
No perceived chance of becoming infected with Covid in next 12 months	58.6 (57.3 - 59.8)	57.8 (54.7 - 60.8)	59.2 (57.4 - 61.0)	1.03 (0.89 - 1.20)	0.66	58.1 (56.0 - 60.2)	0.94 (0.80 - 1.10)	0.42
Vaccinated against COVID-19	10.9 (10.2 - 11.7)	1.80 (1.15 - 2.81)	7.42 (6.54 - 8.41)	4.69 (2.92 - 7.55)	<0.001	19.9 (18.3 - 21.6)	14.7 (9.15 - 23.5)	<0.001

* Odds ratios are adjusted for 5 year age group and site type. For variables limited to 15-19 year olds odds ratios are adjusted for site type only.
