# Supplementary material for: The impact of COVID-19 on sexual risk behaviour for HIV acquisition in east Zimbabwe: An observational study
Source: PLOS Glob Public Health. 2024 Jul 17;4(7):e0003194. doi: 10.1371/journal.pgph.0003194 (PMC11253984; doi:10.1371/journal.pgph.0003194)
Supplement: S7 Table — February -March 2021; April ‐ May 2021; June ‐ July 2021. (PDF) [file pgph.0003194.s011.pdf]

S7 Table. Sexual Risk Behaviours of HIV negative adults aged 15-54 in Manicaland, prior to and during three periods of the Covid-19 pandemic survey.

	Pre-Covid-19	During Covid-19 - Period 1			During Covid-19 - Period 2			During Covid-19 - Period 3		
	% (95%CI)*	% (95%CI)	AOR† (95%CI)	p	% (95%CI)	AOR† (95%CI)	p	% (95%CI)	AOR† (95%CI)	p
**A) Males**										
**15-19yrs**	**N=986**	**N=105**			**N=309**			**N=302**		
Had sexual debut (15-19yrs)	10.8 (8.96 - 12.8)	17.1 (11.0 - 25.7)	1.66 (0.95 - 2.88)	0.07	12.9 (9.63 - 17.2)	1.21 (0.82 - 1.79)	0.34	11.6 (8.43 - 15.7)	1.15 (0.76 - 1.74)	0.51
**15-54yrs started sex**	**N=2050**	**N=313**			**N=874**			**N=776**		
Multiple partners in past 1 month	5.93 (4.97 - 7.06)	3.19 (1.72 - 5.85)	0.49 (0.25 - 0.94)	0.03	5.15 (3.86 - 6.83)	0.79 (0.55 - 1.13)	0.19	4.90 (3.58 - 6.66)	0.88 (0.60 - 1.28)	0.50
Multiple partners in past 12 months	17.8 (16.2 - 19.5)	18.2 (14.3 - 22.9)	1.01 (0.74 - 1.39)	0.93	20.1 (17.6 - 22.9)	1.12 (0.91 - 1.37)	0.29	15.2 (12.8 - 17.9)	0.89 (0.70 - 1.12)	0.31
Concurrent partners	6.62 (5.61 - 7.81)	7.03 (4.66 - 10.5)	1.04 (0.65 - 1.66)	0.88	7.55 (5.97 - 9.50)	1.11 (0.81 - 1.51)	0.52	5.54 (4.13 - 7.39)	0.90 (0.63 - 1.29)	0.56
1 or more non-regular partner in the past year	19.3 (17.6 - 21.0)	19.5 (15.5 - 24.3)	1.02 (0.74 - 1.41)	0.88	22.0 (19.3 - 24.8)	1.18 (0.96 - 1.46)	0.12	18.4 (15.9 - 21.3)	1.08 (0.86 - 1.35)	0.53
Transactional Sex	7.39 (6.31 - 8.64)	8.95 (6.24 - 12.7)	1.26 (0.83 - 1.94)	0.28	7.09 (5.57 - 9.00)	0.97 (0.71 - 1.33)	0.87	5.54 (4.13 - 7.39)	0.79 (0.55 - 1.13)	0.19
STI symptoms in past 12 months	3.12 (2.44 - 3.97)	1.92 (0.86 - 4.21)	0.54 (0.23 - 1.27)	0.16	1.14 (0.62 - 2.11)	0.29 (0.15 - 0.58)	<0.001	1.55 (0.88 - 2.71)	0.54 (0.28 - 1.01)	0.05
**Risk Category‡**	**N=3181**	**N=418**			**N=1233**			**N=1122**		
Not sexually active	30.4 (28.9 - 32.0)	25.1 (21.2 - 29.5)	0.66 (0.45 - 0.97)	0.04	29.1 (26.6 - 31.7)	1.01 (0.80 - 1.29)	0.91	30.8 (28.2 - 33.6)	1.02 (0.79 - 1.31)	0.88
Low risk	50.7 (48.9 - 52.5)	52.4 (47.6 - 57.2)	0.96 (0.74 - 1.25)	0.78	49.6 (46.8 - 52.3)	0.88 (0.74 - 1.05)	0.16	52.0 (49.1 - 55.0)	1.00 (0.84 - 1.20)	0.96
Medium risk	5.49 (4.70 - 6.41)	7.89 (5.66 - 10.9)	1.41 (0.94 - 2.11)	0.09	5.76 (4.59 - 7.21)	1.04 (0.77 - 1.40)	0.80	4.37 (3.31 - 5.73)	0.82 (0.58 - 1.15)	0.25
High risk	13.4 (12.3 - 14.7)	14.6 (11.5 - 18.3)	1.14 (0.84 - 1.54)	0.40	15.6 (13.7 - 17.7)	1.16 (0.95 - 1.41)	0.14	12.7 (10.9 - 14.8)	1.07 (0.86 - 1.32)	0.55
**B) Females**										
**15-19yrs**	**N=1112**	**N=125**			**N=351**			**N=258**		
Had sexual debut (15-19yrs)	29.7 (27.1 - 32.4)	20.0 (13.8 - 28.0)	0.57 (0.36 - 0.91)	0.02	19.7 (15.8 - 24.2)	0.57 (0.43 - 0.77)	<0.001	21.3 (16.7 - 26.8)	0.69 (0.50 - 0.96)	0.03
**15-54yrs started sex**	N=3172	N=516			N=1428			N=864		
Multiple partners in past 1 month	1.06 (0.76 - 1.49)	0.58 (0.19 - 1.79)	0.52 (0.16 - 1.72)	0.29	0.77 (0.43 - 1.39)	0.80 (0.40 - 1.59)	0.52	1.27 (0.71 - 2.29)	1.00 (0.50 - 2.01)	0.99
Multiple partners in past 12 months	6.07 (5.29 - 6.95)	2.33 (1.32 - 4.05)	0.37 (0.21 - 0.67)	0.00	3.29 (2.48 - 4.35)	0.56 (0.40 - 0.78)	0.00	4.05 (2.92 - 5.59)	0.65 (0.45 - 0.95)	0.03
Concurrent partners	1.03 (0.73 - 1.45)	0.39 (0.10 - 1.54)	0.34 (0.08 - 1.44)	0.14	0.77 (0.43 - 1.39)	0.78 (0.39 - 1.57)	0.50	1.27 (0.71 - 2.29)	1.05 (0.52 - 2.12)	0.89
1 or more non-regular partner in the past year	8.30 (7.40 - 9.30)	6.78 (4.91 - 9.31)	0.92 (0.63 - 1.33)	0.64	6.93 (5.72 - 8.37)	0.95 (0.74 - 1.21)	0.67	8.33 (6.66 - 10.4)	1.11 (0.84 - 1.48)	0.45
Transactional Sex	6.90 (6.06 - 7.84)	3.68 (2.36 - 5.71)	0.55 (0.34 - 0.90)	0.02	5.60 (4.52 - 6.92)	0.83 (0.64 - 1.09)	0.18	6.37 (4.92 - 8.20)	0.92 (0.67 - 1.25)	0.60
Age disparate relationship 5 years or more	61.2 (59.5 - 62.9)	61.5 (57.2 - 65.7)	1.10 (0.90 - 1.34)	0.35	62.1 (59.6 - 64.6)	1.11 (0.98 - 1.27)	0.11	61.3 (58.0 - 64.5)	1.07 (0.91 - 1.25)	0.43
Age disparate relationship 10 years or more	21.8 (20.4 - 23.3)	21.7 (18.3 - 25.5)	1.00 (0.80 - 1.27)	0.97	22.7 (20.6 - 25.0)	1.03 (0.89 - 1.21)	0.66	19.0 (16.5 - 21.8)	0.84 (0.69 - 1.02)	0.08
STI symptoms in past 12 months	7.54 (6.66 - 8.53)	9.50 (7.25 - 12.4)	1.26 (0.91 - 1.75)	0.17	7.91 (6.62 - 9.43)	1.05 (0.82 - 1.33)	0.71	6.02 (4.61 - 7.82)	0.89 (0.65 - 1.21)	0.45
**Risk Category‡**	**N=4135**	**N=635**			**N=1785**			**N=1121**		
Not sexually active	18.8 (17.8 - 20.0)	18.7 (15.9 - 22.0)	1.31 (0.95 - 1.81)	0.10	20.0 (18.2 - 21.9)	1.60 (1.30 - 1.97)	<0.001	22.9 (20.6 - 25.5)	1.53 (1.20 - 1.95)	0.001
Low risk	70.1 (68.7 - 71.5)	72.9 (69.3 - 76.2)	1.01 (0.79 - 1.28)	0.96	71.3 (69.2 - 73.4)	0.89 (0.76 - 1.04)	0.15	66.9 (64.1 - 69.6)	0.80 (0.67 - 0.96)	0.02
Medium risk	4.32 (3.72 - 5.01)	2.83 (1.79 - 4.46)	0.67 (0.41 - 1.10)	0.11	3.14 (2.42 - 4.06)	0.70 (0.52 - 0.96)	0.03	3.75 (2.78 - 5.03)	0.90 (0.63 - 1.27)	0.54
High risk	6.73 (6.00 - 7.55)	5.51 (3.98 - 7.59)	0.85 (0.59 - 1.22)	0.37	5.55 (4.57 - 6.71)	0.85 (0.67 - 1.08)	0.18	6.42 (5.13 - 8.02)	1.02 (0.77 - 1.34)	0.90

* In the cross-sectional analysis of the pre-covid survey, proportions are weighted to account for age bias in selection

† Odds ratios are adjusted for 5 year age group and site type. For variables limited to 15-19 year olds odds ratios are adjusted for site type only. AORs are compared to the Pre-Covid-19 survey.

‡ Low risk =no risk behaviors, Medium risk = concurrent partners, more than one partner in the past 12 months, transactional sex, High risk = non-regular partners
