# Supplementary material for: The impact of COVID-19 on sexual risk behaviour for HIV acquisition in east Zimbabwe: An observational study
Source: PLOS Glob Public Health. 2024 Jul 17;4(7):e0003194. doi: 10.1371/journal.pgph.0003194 (PMC11253984; doi:10.1371/journal.pgph.0003194)
Supplement: S1 Text — Further detail regarding the source of data used in this analysis. (PDF) [file pgph.0003194.s012.pdf]

S1 Text. Data source

In each round of the survey, an initial household census was carried out in each study site and individuals aged 15 years and above resident in the enumerated households were eligible and invited to participate in individual interviews. Data from Pre-Covid-19 survey were used as the frame for the initial household census in the During-Covid-19 survey.

'Pre-Covid-19' survey: In the 2018/19 survey round, the eight study sites were covered one at a time over an 18-month period. Younger people (females aged 15-24 years and males aged 15-29 years) resident in all households were eligible to participate but older people were eligible only if they were resident in a random sample of two-thirds of households. 20 Interviews were conducted face-to-face at participants' households and included questions on socio-demographic characteristics and SRBs for HIV acquisition. Data on participants' HIV infection status were obtained through provider-initiated HIV testing and counselling (PITC) or from laboratory testing on dried blood spots (DBS) where participants declined PITC but gave written informed consent to provide DBS specimens for the study.

'During Covid-19' survey: In the 2021 survey, the eight study sites were divided into two equal groups with the same mix of socio-economic locations and enumerated in two consecutive phases over a combined 5-month period to provide timely information for local policy-makers. For people of all ages, eligibility for the individual interview was restricted to those resident in the random sample of two-thirds of households. The survey was limited by Covid-19 safety measures. Interview procedures were adapted to take place over the telephone and self-reported data were collected on HIV testing and infection status. Additional questions were added to the individual interview questionnaire to provide data on participants' perceptions, experiences, and responses to Covid-19.
