# Supplementary material for: The impact of COVID-19 on sexual risk behaviour for HIV acquisition in east Zimbabwe: An observational study
Source: PLOS Glob Public Health. 2024 Jul 17;4(7):e0003194. doi: 10.1371/journal.pgph.0003194 (PMC11253984; doi:10.1371/journal.pgph.0003194)
Supplement: S2 Text — Supplementary detail regarding definitions of study measures including a) HIV status; b) Household wealth; c) Education; d) Risk categories. (PDF) [file pgph.0003194.s013.pdf]

S2 Text. Definitions of study measures.

Demographic characteristics of participants including age-group, marital status, education, and employment as well as drug and alcohol use were obtained from questions included in both rounds of the survey (S1 Table). In the during-Covid-19 survey, additional questions were added to capture awareness of Covid-19 and its symptoms (shortness of breath, sore throat, loss of sense of smell and loss of sense of taste), prevention behaviours such as isolating, missing school and getting vaccinated as well as perceived risk of becoming infected with Covid-19 (S2 Table).

Sexual Risk Behaviour (SRB) variables (S3 Table) were defined as having had sexual debut for participants aged 15-19 years; and for those aged 15-54 years who reported having started sex: having had more than one sexual partner in the past month and in the past 12 months (two variables); having concurrent partners at the time of interview; having one or more non-regular partners in the past 12 months; having had transactional sex in the past month with any of the three most recent sexual partners; and, for women only, having had an age-disparate sexual relationship with one of their last three partners in the past 12 months (age-disparate relationships defined as being partnerships with someone ≥10 years older). A variable for having experienced symptoms of a sexually transmitted infection (males were asked about penile discharge and/or dysuria, and females were asked vaginal discharge, dysuria or genital sores) in the past 12 months was also created as a further indicator of having engaged in recent SRB.

A) HIV Status

In the pre-Covid-19 survey round, participants HIV infection status was established using the results from provider-initiated testing and counselling (PITC) and, where PITC results were not available, from laboratory testing.[1] In the during-Covid-19 round, HIV status infection was determined using test results from the pre-Covid-19 survey round updated with self-reports: 1) for individuals who participated and tested HIV-negative in the pre-Covid-19 round, of results from any HIV testing received between rounds; or 2) for individuals newly enrolled in the survey, of results of their most recent HIV test. Participants in the during-Covid-19 survey round who had never had an HIV test or who were HIV-negative in the pre-Covid-19 survey and had not had a test since then were assumed to be uninfected.

B) Household Wealth

Household wealth was estimated for each individual by assessing asset ownership within their household and attributing a score as described by Schur and colleagues.[2] Each asset variable was transformed into scores between 0 and 1. The values of each asset variable were then summed and divided by the total number of assets giving an overall score between 0 and 1. Equally spaced cut-offs (0, 0.2, 0.4, 0.6, 0.8) were used to categorise the overall wealth of a household into five groups.

C) Education

Participants were asked what their highest level of education was with possible answers being; None/Primary, Secondary or Tertiary. However, a discrepancy was noted between the two rounds with the answers of many cohort participants who completed both the pre-Covid Survey and during-Covid Survey reporting a higher level of education in the pre-Covid-19 survey. To mitigate the impact of this inconsistent coding, answers were updated for those who participated in both rounds of the survey if they reported a higher level of education as their 'highest level' pre-covid. In these cases, the participants' response to the question in the 'during Covid-19 survey' was updated to match that given in the pre-covid survey.

D) Risk Categories

Risk categories were created to assess overall changes in SRB. High risk for HIV acquisition was defined as reporting two or more non-regular partners in the past year. Participants were categorised as medium risk if they reported multiple partners in the past year or having engaged in transactional sex in the past 1 month with any of the 3 most recent sexual partner but did not fulfil any of the high-risk criteria. Low risk was defined as having started sex but not fulfilling any other risk criteria.

References

1. Rao A, Moorhouse L, Maswera R, Dadirai T, Mandizvidza P, Nyamukapa C, et al. Status of the HIV epidemic in Manicaland, east Zimbabwe prior to the outbreak of the COVID-19 pandemic. PloS One. 2022;17(9):e0273776.

2. Schur N, Mylne A, Mushati P, Takaruza A, Ward H, Nyamukapa C, et al. The effects of household wealth on HIV prevalence in Manicaland, Zimbabwe - a prospective household census and population-based open cohort study. J Int AIDS Soc. 2015;18:20063.
