# Supplementary material for: The impact of COVID-19 on sexual risk behaviour for HIV acquisition in east Zimbabwe: An observational study
Source: PLOS Glob Public Health. 2024 Jul 17;4(7):e0003194. doi: 10.1371/journal.pgph.0003194 (PMC11253984; doi:10.1371/journal.pgph.0003194)
Supplement: S3 Text — Explanation of analysis shown in S4–S7 Tables. (PDF) [file pgph.0003194.s014.pdf]

S3 Text. Periodic cross-sectional analysis.

Data from the During-Covid-19 survey were split into three periods for analysis reflecting the progression of the Covid-19 pandemic in Zimbabwe. Period 1 covered February and March 2021, Period 2: April and May 2021 and Period 3: June and July 2021.

The results of this analysis can be found in Tables S4-S7.
